# Costs of biomarker testing in advanced non‐small cell lung cancer: a global study comparing next‐generation sequencing and single‐gene testing

**DOI:** 10.1002/2056-4538.70018

**Published:** 2025-03-07

**Authors:** Umberto Malapelle, Chien‐Chin Chen, Enrique de Álava, Paul Hofman, Daniel Kazdal, Tae‐Jung Kim, Tony Kiat Hon Lim, Aleš Ryška, Angelica A Saetta, Ed Schuuring, Giancarlo Troncone, Michele Biscuola, Yi‐Lin Chen, Gek San Tan, Charles Hugo Marquette, Maria Michelli, Arja ter Elst, Hana Vošmiková, Joshua Kapp, Sebastian Gonzalez‐McQuire, Andromachi Giannopoulou, Jean Marie Franzini, Victoria Lucia Rabsiun Aramburu, Anna Baggi, Albrecht Stenzinger

**Affiliations:** ^1^ Department of Public Health University of Naples Federico II Naples Italy; ^2^ Department of Pathology Ditmanson Medical Foundation Chia‐Yi Christian Hospital Chia‐Yi Taiwan; ^3^ Virgen del Rocio University Hospital, Institute of Biomedicine of Sevilla (IBiS), CSIC University of Sevilla, CIBERONC Seville Spain; ^4^ Department of Normal and Pathological Cytology and Histology, School of Medicine University of Seville Seville Spain; ^5^ Laboratory of Clinical and Experimental Pathology, IHU RespirERA, FHU OncoAge, Biobank 0033‐00025 University Côte d'Azur Nice France; ^6^ Institute of Pathology University Hospital Heidelberg, Center for Personalized Medicine (ZPM) Heidelberg Germany; ^7^ Translational Lung Research Center Heidelberg (TLRC‐H) Member of the German Center for Lung Research (DZL) Heidelberg Germany; ^8^ Department of Hospital Pathology, Yeouido St. Mary's Hospital, College of Medicine The Catholic University of Korea Seoul Republic of Korea; ^9^ Division of Pathology Singapore General Hospital Singapore; ^10^ The Fingerland Department of Pathology Charles University Faculty of Medicine and University Hospital Hradec Králové Czech Republic; ^11^ Molecular Diagnostic Unit, First Department of Pathology, School of Medicine National and Kapodistrian University of Athens Athens Greece; ^12^ Department of Pathology and Medical Biology University Medical Center Groningen, University of Groningen Groningen The Netherlands; ^13^ Department of Pathology, National Cheng Kung University Hospital, College of Medicine National Cheng Kung University Tainan Taiwan; ^14^ Department of Thoracic Oncology University Cote d'Azur, CHU de Nice, IHU RespirERA Nice France; ^15^ Amgen (Europe) Rotkreuz Switzerland; ^16^ Life Sciences Division BIP Consulting Milan Italy

**Keywords:** cost comparison, next‐generation sequencing, NSCLC, precision medicine, predictive biomarker, single‐gene testing

## Abstract

Current European/US guidelines recommend that molecular testing in advanced non‐small cell lung cancer (aNSCLC) be performed using next‐generation sequencing (NGS). However, the global uptake of NGS is limited, largely owing to reimbursement constraints. We compared real‐world costs of NGS and single‐gene testing (SGT) in nonsquamous aNSCLC. This observational study was conducted across 10 pathology centers in 10 different countries worldwide. Biomarker data collected via structured questionnaires (1 January–31 December 2021) were used to feed micro‐costing analyses for three scenarios [‘Starting Point’ (SP; 2021–2022), ‘Current Practice’ (CP; 2023–2024), and ‘Future Horizons’ (FH; 2025–2028)] in both a real‐world model, comprising all biomarkers tested by each center, and a standardized model, comprising the same sets of biomarkers across centers. Testing costs (including retesting) encompassed personnel costs, consumables, equipment, and overheads. Overall, 4,491 patients with aNSCLC were evaluated. Mean per‐patient costs decreased for NGS relative to SGT over time, with real‐world model costs 18% lower for NGS than for SGT in the SP scenario, and 26% lower for NGS than for SGT in the CP scenario. Mean per‐biomarker costs also decreased over time for NGS relative to SGT. In the standardized model, the tipping point for the minimum number of biomarkers required for NGS to result in cost savings (per patient) was 10 and 12 in the SP and CP scenarios, respectively. Retesting had a negligible impact on cost analyses, and results were robust to variation in cost parameters. This study provides robust real‐world global evidence for cost savings with NGS‐based panels over SGT to evaluate predictive biomarkers in nonsquamous aNSCLC when the number of biomarkers to be tested exceeds 10. Widespread adoption of NGS may enable more efficient use of limited healthcare resources.

## Introduction

Non‐small cell lung cancer (NSCLC) represents approximately 80% of all lung cancers [1]; the most common histopathological subtype is adenocarcinoma [2]. Over the past two decades, the identification of actionable genomic alterations in patients with advanced nonsquamous NSCLC (aNSCLC) has led to the development of multiple targeted therapies as part of the continuous effort to improve patient outcomes [3], resulting in increasingly complex molecular diagnoses [4].

To date, targeted agents are approved by the European Medicines Agency and US Food and Drug Administration (FDA) as first‐ or later‐line treatment in patients with nonsquamous aNSCLC who have genomic alterations in *EGFR*, *ALK*, *ROS1*, *BRAF*, *NTRK*, *MET*, *RET*, *KRAS*, and *ERBB2*/*HER2* [5, 6, 7]. The most current international guidelines, developed by the European Society for Medical Oncology (ESMO) and National Comprehensive Cancer Network® (NCCN®), recommend testing the following biomarkers in patients with nonsquamous aNSCLC: *EGFR* mutations, *ALK* fusions, *ROS1* fusions, *BRAF* p.V600E mutations, *NTRK1*/*2*/*3* fusions, *MET* exon 14 skipping mutations, *MET* amplifications, *RET* fusions, *KRAS* p.G12C mutations, *ERBB2*/*HER2* mutations, and programmed cell death ligand 1 (PD‐L1) expression [6, 7, 8].

ESMO and NCCN Clinical Practice Guidelines in Oncology (NCCN Guidelines®) recommend that testing be performed using next‐generation sequencing (NGS) [6, 7, 9]. Unlike the sequential assay approach used with single‐gene testing (SGT), NGS uses a panel‐based approach to evaluate multiple biomarkers in parallel. Compared with SGT, the potential benefits of NGS are multifold, and include faster turnaround time [10, 11, 12, 13], greater sensitivity [14], and more efficient use of limited tissue samples [14]. Indeed, upfront molecular testing with SGT versus multiplex comprehensive genomic profiling has been shown to deplete available tissue, thus preventing complete testing of guideline‐recommended biomarkers in the case of negative SGT results [15].

Nevertheless, widespread access to NGS testing for aNSCLC is limited, with significant heterogeneity within and between countries, largely as a result of reimbursement constraints [16, 17, 18]. For example, many centers across Europe still use SGT as the primary means of detecting *EGFR* mutations [19]. In addition, there are few data regarding the clinical availability, funding, and uptake of NGS globally [20, 21, 22].

Although individual SGTs are typically less expensive than NGS, NGS may offer overall cost savings as the number of actionable biomarkers increases. However, data from most studies designed to compare costs of NGS and SGT are limited by the use of hypothetical patient populations and/or patients tested in single centers or countries [12, 13, 23, 24, 25, 26, 27, 28, 29]. The present study investigated biomarker testing strategies for patients with aNSCLC at 10 global centers of excellence with expertise across multiple testing modalities. Micro‐costing was employed to compare the costs of NGS versus SGT over time using both real‐world and standardized models.

## Materials and methods

### Ethics statement

All data were handled anonymously by the respective centers, and procedures were followed in compliance with the Declaration of Helsinki. No patient‐level data of a personal or sensitive nature were included in this research, and therefore approvals from Institutional Review Boards or Independent Ethics Committees were not required for this study.

### Study design

**Figure 1:**
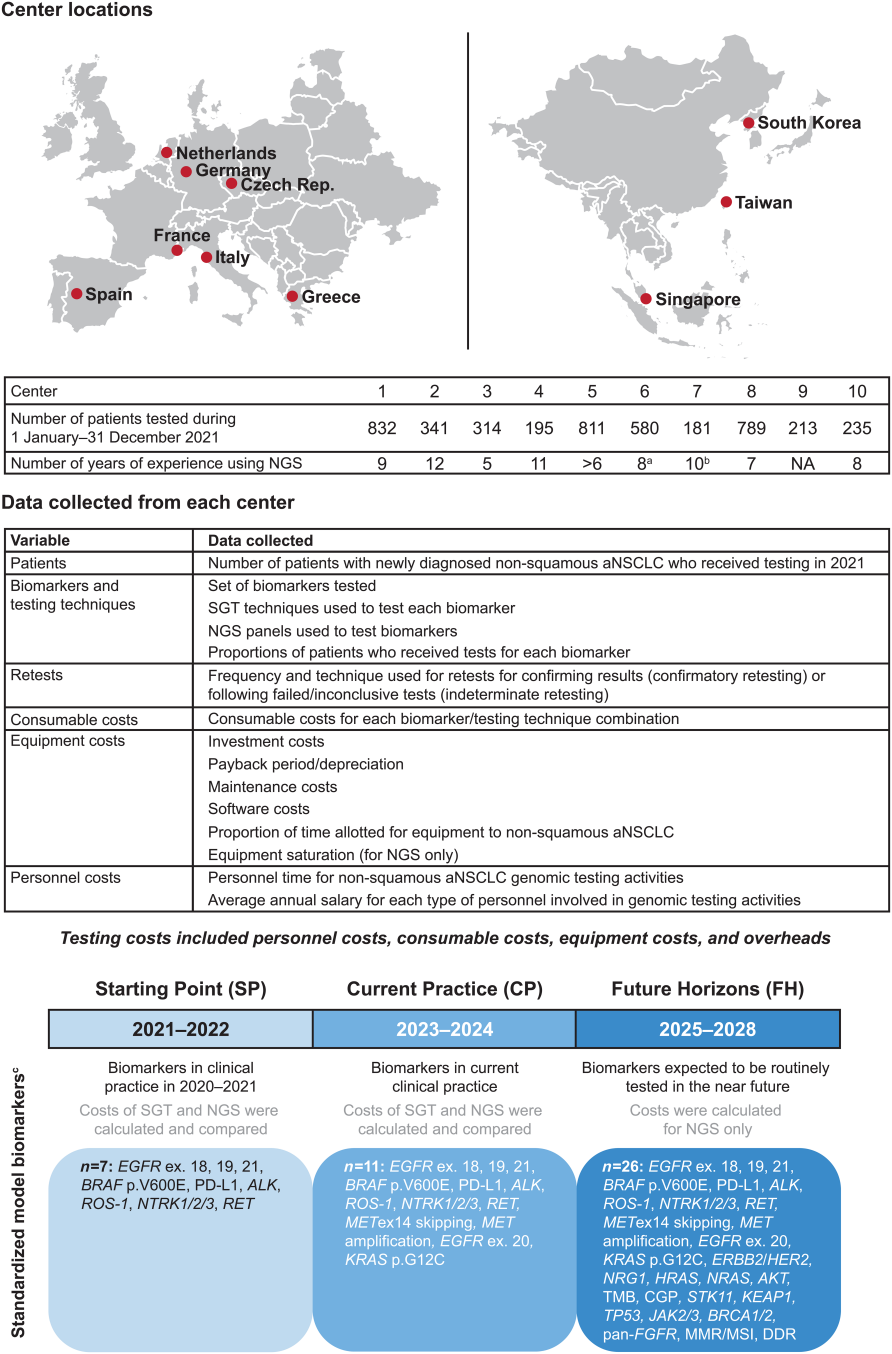
Study design. Map created using Power‐user SAS. ^a^Center 6 used NGS partially since 2015. ^b^Center 7 used NGS for predictive testing (for all cancer types) since 2002, as well as for diagnostic testing since 2013. ^c^Biomarkers included in the standardized model were the same across centers; biomarkers included in the real‐world model differed between centers. *AKT*, protein kinase B; *ALK*, anaplastic lymphoma kinase; *BRAF*, v‐raf murine sarcoma viral oncogene homolog B1; *BRCA*, BReast CAncer gene; CGP, comprehensive genomic profiling; CP, Current Practice; DDR, DNA damage response; *EGFR*, epidermal growth factor receptor; *ERBB2*, erb‐b2 receptor tyrosine kinase 2; *FGFR*, fibroblast growth factor receptor; FH, Future Horizons; FISH, fluorescence *in situ* hybridization; *HER2*, human epidermal growth factor receptor 2; *HRAS*, Harvey rat sarcoma viral oncogene homolog; IHC, immunohistochemistry; ISH, *in situ* hybridization; *JAK*, Janus kinase; *KEAP1*, Kelch‐like ECH‐associated protein 1; *KRAS*, Kirsten rat sarcoma viral oncogene homologue; *MET*, mesenchymal epithelial transition factor receptor; MMR, mismatch repair; MSI, microsatellite instability; NA, not available; NGS, next‐generation sequencing; *NRAS*, neuroblastoma RAS viral oncogene homolog; *NRG1*, neuregulin 1; *NTRK*, neurotrophic tyrosine receptor kinase; PCR, polymerase chain reaction; PD‐L1, programmed cell death ligand 1; *PIK3CA*, phosphatidylinositol‐4,5‐bisphosphate 3‐kinase, catalytic subunit alpha; *RET*, rearranged during transfection; *ROS1*, c‐ros oncogene 1; RT, real‐time; SGT, single‐gene testing; SP, Starting Point; *STK11*, serine/threonine kinase 11; *TP53*, tumor protein p53.

This international, cross‐sectional, observational study was conducted across 10 centers, one in each of the following countries: Czech Republic, France, Germany, Greece, Italy, South Korea, Singapore, Spain, the Netherlands, and Taiwan (Figure 1). Participating centers performed molecular profiling for patients with nonsquamous aNSCLC, and had access to and experience of SGT and NGS, enabling unbiased comparison of both methodologies within centers. Data on the evaluated biomarkers, testing techniques, retesting frequencies, and related costs associated with patients with nonsquamous aNSCLC who had received molecular testing between 1 January and 31 December 2021 were collected retrospectively from each center using a structured questionnaire (see supplementary material, File S1). These data were collected according to three temporal scenarios: ‘Starting Point’ (SP), ‘Current Practice’ (CP), and ‘Future Horizons’ (FH) (see Figure 1). For each scenario, two different models were used to determine costs of SGT and NGS: a real‐world model, comprising the biomarkers and SGT/NGS techniques reported by each center across the three scenarios, allowing insight into recent, current, and future real‐world practice; and a standardized model, comprising the same predefined set of biomarkers and SGT testing techniques across centers in each scenario, enabling inter‐site cost comparison. Details of the biomarkers included in each model are given in Figure 1 and in the Supplementary materials and methods. A list of abbreviations is provided as supplementary material, Table S1.

### Data collection

The types of data collected from each center are summarized in Figure 1. Data were reviewed by the study team and validated by the principal investigator of each center. Details of how data (including missing data) were handled are given in the Supplementary materials and methods.

### Data analysis

Data on resource use and costs were used to feed micro‐costing analyses that compared the total costs of NGS and SGT in both models for the SP and CP scenarios. Total costs included personnel costs, consumable costs, equipment costs, and overheads (Figure 1). Details of cost calculations are given in the Supplementary materials and methods. For the FH scenario, only NGS costs were determined, owing to the large number of biomarkers included for which SGT would likely not be practical or economically viable. In both models, costs specific to each center were applied; for the standardized model, only costs related to the fixed set of biomarkers and associated detection techniques were included in cost calculations. In both models, analyses accounted for retesting.

In the standardized model, the tipping point of the minimum number of biomarkers required for per‐patient costs of NGS to be less than those of SGT was calculated for the SP and CP scenarios. In brief, the SGT per‐patient cost was divided by the number of biomarkers to obtain the SGT cost per patient as a linear function of the number of biomarkers; the NGS per‐patient cost was assumed to be constant, independent of the number of biomarkers tested.

In the real‐world model, the effect of including retests in cost calculations on the difference in total costs per patient of NGS versus SGT was evaluated in the SP and CP scenarios. In the standardized model, the rate of retesting was held constant across the three scenarios to enable inter‐center cost comparisons [SGT: *ALK*, 4%; *ROS1*, 2%; *NTRK1*/*2*/*3*, 1% (all confirmatory retests); NGS: 0%].

For both models, a deterministic sensitivity analysis (DSA) was performed to determine the impact of altering individual cost parameters by ±20% on the difference in total annual testing costs of NGS versus SGT in the SP and CP scenarios.

## Results

### Biomarker testing frequencies and techniques

Overall, tumor samples from 4,491 patients with nonsquamous aNSCLC were tested from 1 January to 31 December 2021 across the centers (per‐center range: *n* = 181–832) (Figure 1), with biomarker testing and cost data for these patients used to feed the micro‐costing analyses. The biomarkers tested in the SP, CP, and FH scenarios are shown in supplementary material, Table S2 (real‐world model) and Figure 1 (standardized model). In the real‐world model, the number of biomarkers tested across centers increased over time [mean (range): SP, 14 (11–21); CP, 17 (11–26); FH, 25 (17–27)]. In the standardized model, the number of biomarkers included in the SP, CP, and FH scenarios was 7, 11, and 26, respectively.

SGT and NGS techniques/panels used in the real‐world model were reported by each center (supplementary material, Tables S3 and S4). SGT techniques included Sanger sequencing, immunohistochemistry, *in situ* hybridization, fluorescence *in situ* hybridization (FISH), and polymerase chain reaction (PCR). Up to six different DNA/RNA NGS panels were used across centers. In the standardized model, the following SGT techniques were used: PCR‐based techniques for *EGFR* exons 18, 19, 20, and 21, *BRAF* p.V600E, *MET* exon 14 skipping, and *KRAS* p.G12C; FISH for *RET*, and for *MET* amplifications; and immunohistochemistry for PD‐L1, *ALK*, *ROS1*, and *NTRK1*/*2*/*3*. The NGS techniques/panels reported by each center in the real‐world model were also applied to the standardized model.

The mean number of machine runs carried out per patient for SGT and NGS in each scenario and in each center are shown in supplementary material, Figure S1 (real‐world model) and supplementary material, Figure S2 (standardized model).

### Comparison of SGT and NGS testing costs

#### Real‐world model

##### Per‐patient cost comparison

**Figure 2:**
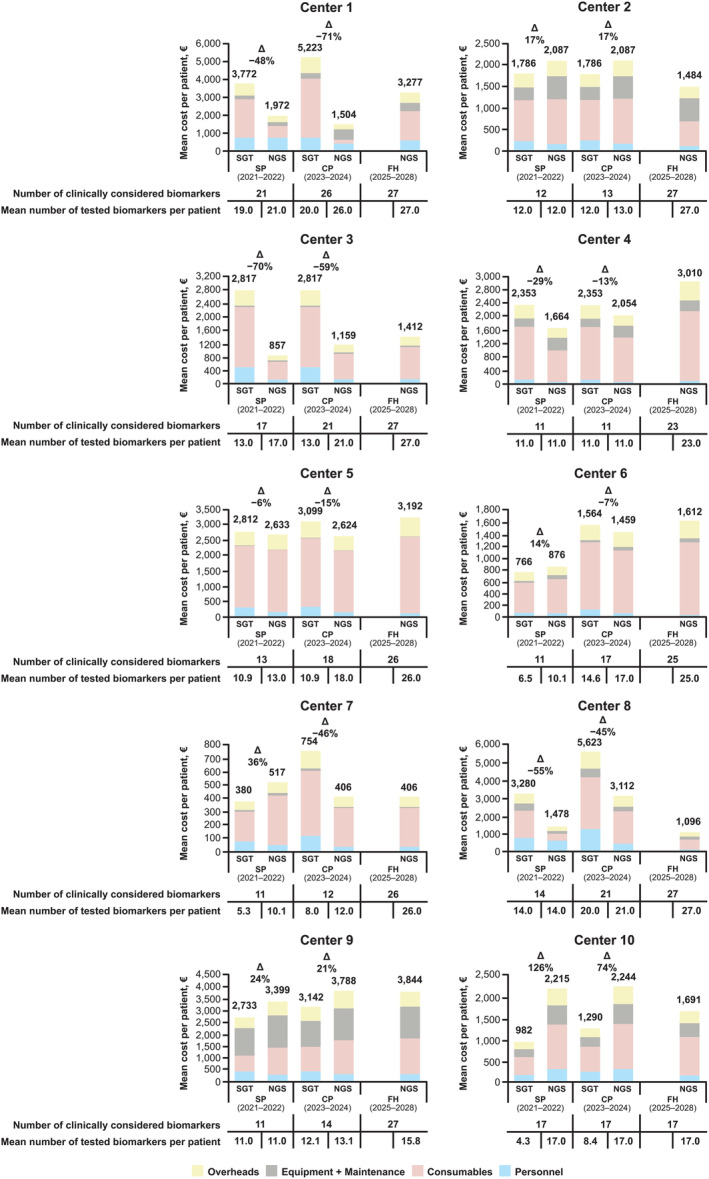
Mean cost per patient for SGT and NGS in the real‐world model. CP, Current Practice; FH, Future Horizons; NGS, next‐generation sequencing; SGT, single‐gene testing, SP, Starting Point.

The largest proportion of per‐patient SGT and NGS costs in the real‐world model were related to consumables (Figure 2). On average, per‐patient costs were lower for NGS than for SGT in the SP and CP scenarios, with greater cost savings with NGS versus SGT observed in the CP scenario (Table 1A and Figure 2). In the SP scenario, per‐patient costs were, on average, 18% (€398) lower for NGS than SGT [center mean (range), €1,770 (€517–3,399) versus €2,168 (€380–3,772), respectively], with costs lower for NGS in 5/10 centers. In the CP scenario, per‐patient costs were, on average, 26% (€721) lower for NGS than SGT [center mean (range), €2,044 (€406–3,788) versus €2,765 (€754–5,623), respectively], with costs lower for NGS in 7/10 centers. In the FH scenario, the mean per‐patient cost of NGS was €2,102 (center range, €406–3,844) (Table 1A).

**Table 1:** Cost differences for NGS versus SGT in (A) the real‐world model and (B) the standardized model

(A)																
	SP						CP						FH			
	Cost per patient			Cost per biomarker			Cost per patient			Cost per biomarker			Cost per patient		Cost per biomarker	
Center	SGT, €	NGS, €	Δ NGS versus SGT, € (%)	SGT, €	NGS, €	Δ NGS versus SGT, € (%)	SGT, €	NGS, €	Δ NGS versus SGT, € (%)	SGT, €	NGS, €	Δ NGS versus SGT, € (%)	SGT, €	NGS, €	SGT, €	NGS, €
1	3,772	1,972	−1,800 (−48)	199	94	−105 (−53)	5,223	1,504	−3,719 (−71)	261	58	−203 (−78)	–	3,277	–	121
2	1,786	2,087	301 (17)	149	174	25 (17)	1,786	2,087	301 (17)	149	161	12 (8)	–	1,484	–	55
3	2,817	857	−1,960 (−70)	217	50	−167 (−77)	2,817	1,159	−1,658 (−59)	217	55	−162 (−75)	–	1,412	–	55
4	2,353	1,664	−689 (−29)	214	151	−63 (−29)	2,353	2,054	−299 (−13)	214	187	−27 (−13)	–	3,010	–	131
5	2,812	2,633	−179 (−6)	258	203	−55 (−21)	3,099	2,624	−475 (−15)	284	146	−138 (−49)	–	3,192	–	123
6	766	876	110 (14)	118	87	−31 (−26)	1,564	1,459	−105 (−7)	107	86	−21 (−20)	–	1,612	–	64
7	380	517	137 (36)	72	51	−21 (−29)	754	406	−348 (−46)	94	34	−60 (−64)	–	406	–	16
8	3,280	1,478	−1,802 (−55)	234	106	−128 (−55)	5,623	3,112	−2,511 (−45)	281	148	−133 (−47)	–	1,096	–	41
9	2,733	3,399	666 (24)	248	309	61 (25)	3,142	3,788	646 (21)	260	289	29 (11)	–	3,844	–	243
10	982	2,215	1,233 (126)	228	130	−98 (−43)	1,290	2,244	954 (74)	154	132	−22 (−14)	–	1,691	–	99
Mean	2,168	1,770	−398 (−18)	194	136	−58 (−29)	2,765	2,044	−721 (−26)	202	130	−72 (−36)	–	2,102	–	95

(B)																
	SP						CP						FH			
	Cost per patient			Cost per biomarker			Cost per patient			Cost per biomarker			Cost per patient		Cost per biomarker	
Center	SGT, €	NGS, €	Δ NGS versus SGT, € (%)	SGT, €	NGS, €	Δ NGS versus SGT, € (%)	SGT, €	NGS, €	Δ NGS versus SGT, € (%)	SGT, €	NGS, €	Δ NGS versus SGT, € (%)	SGT, €	NGS, €	SGT, €	NGS, €
1	1,449	1,663	214 (15)	207	238	31 (15)	2,177	1,164	−1,013 (−47)	198	106	−92 (−46)	–	3,098	–	119
2	977	1,644	667 (68)	140	235	95 (68)	1,581	1,644	63 (4)	144	149	5 (3)	–	1,108	–	43
3	929	728	−201 (−22)	133	104	−29 (−22)	1,499	1,030	−469 (−31)	136	94	−42 (−31)	–	1,283	–	49
4	2,505	1,478	−1,027 (−41)	358	211	−147 (−41)	3,309	1,834	−1,475 (−45)	301	167	−134 (−45)	–	2,617	–	101
5	1,641	2,892	1,251 (76)	234	413	179 (76)	2,338	2,881	543 (23)	213	262	49 (23)	–	3,602	–	139
6	1,003	789	−214 (−21)	143	113	−30 (−21)	1,385	1,360	−25 (−2)	126	124	−2 (−2)	–	1,513	–	58
7	629	524	−105 (−17)	90	75	−15 (−17)	1,200	431	−769 (−64)	109	39	−70 (−64)	–	431	–	17
8	1,255	1,318	63 (5)	179	188	9 (5)	1,986	1,454	−532 (−27)	181	132	−49 (−27)	–	1,040	–	40
9	1,931	2,868	937 (49)	276	410	134 (49)	2,444	3,010	566 (23)	222	274	52 (23)	–	3,149	–	121
10	1,389	2,244	855 (62)	198	321	123 (62)	2,470	2,244	−226 (−9)	225	204	−21 (−9)	–	1,691	–	65
Mean	1,371	1,615	244 (18)	196	231	35 (18)	2,039	1,705	−334 (−16)	186	155	−31 (−17)	–	1,953	–	75

Green cells represent lower costs for NGS versus SGT; orange cells represent higher costs for NGS versus SGT.

CP, Current Practice; FH, Future Horizons; NGS, next‐generation sequencing; SGT, single‐gene testing; SP, Starting Point.

##### Per‐biomarker cost comparison

In the real‐world model, per‐biomarker costs were lower for NGS than for SGT in the SP and CP scenarios, with greater cost savings with NGS versus SGT seen in the CP scenario (Table 1A and supplementary material, Figure S3). In the SP scenario, per‐biomarker costs were, on average, 29% (€58) lower for NGS than SGT [center mean (range), €136 (€50–309) versus €194 (€72–258), respectively]. In the CP scenario, per‐biomarker costs were, on average, 36% (€72) lower for NGS than SGT [center mean (range) €130 (€34–289) versus €202 (€94–284), respectively]. Per‐biomarker costs were lower for NGS in most centers (8/10) in both the SP and CP scenarios. Mean NGS costs per biomarker decreased over time (€136, €130, and €95 in the SP, CP, and FH scenarios, respectively) (Table 1A).

#### Standardized model

##### Per‐patient cost comparison

**Figure 3:**
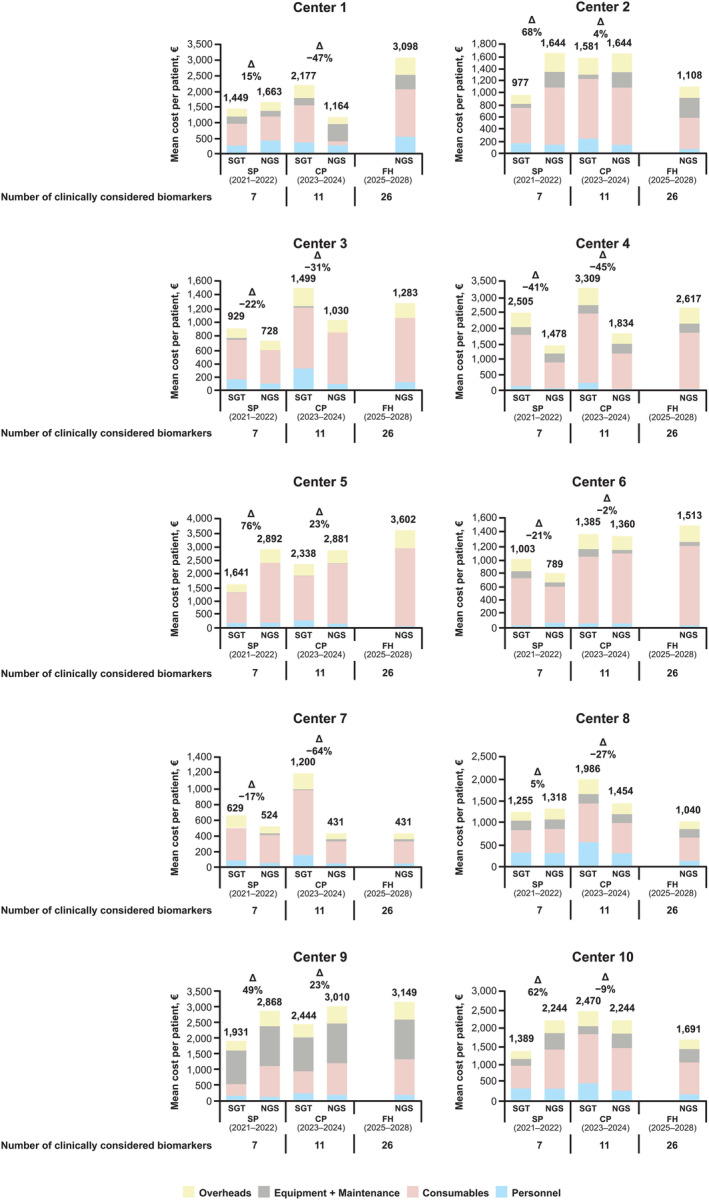
Mean cost per patient for SGT and NGS in the standardized model. CP, Current Practice; FH, Future Horizons; NGS, next‐generation sequencing; SGT, single‐gene testing; SP, Starting Point.

In the standardized model (which included the same fixed sets of biomarkers across each center), as per the real‐world model, the largest proportion of per‐patient testing costs was related to consumables (Figure 3). On average, per‐patient costs were higher for NGS than for SGT in the SP scenario, but lower in the CP scenario (Table 1B and Figure 3). In the SP scenario, per‐patient costs were, on average, 18% (€244) higher for NGS than SGT [center mean (range), €1,615 (€524–2,892) versus €1,371 (€629–2,505), respectively], with costs higher for NGS in 6/10 centers. Conversely, in the CP scenario, per‐patient costs were, on average, 16% (€334) lower for NGS than SGT [center mean (range), €1,705 (€431–3,010) versus €2,039 (€1,200–3,309), respectively], with costs lower for NGS in 7/10 centers. In the FH scenario, the mean per‐patient cost of NGS was €1,953 (center range, €431–3,602) (Table 1B).

The tipping point, in terms of the mean number of biomarkers required to be tested for NGS to be less expensive per patient than SGT, was 10 (center range, 5–15) in the SP scenario, and 12 (center range, 4–18) in the CP scenario (supplementary material, Table S5).

##### Per‐biomarker cost comparison

In the standardized model, as per the real‐world model, per‐biomarker costs of NGS decreased relative to those of SGT in the CP versus SP scenario (Table 1B and supplementary material, Figure S4). In the SP scenario, per‐biomarker costs were, on average, 18% (€35) higher for NGS than SGT [center mean [range], €231 (€75–413) versus €196 (€90–358), respectively], with costs higher for NGS in 6/10 centers. Conversely, in the CP scenario, per‐biomarker costs were, on average, 17% (€31) lower for NGS than for SGT [center mean (range), €155 (€39–274) versus €186 (€109–301), respectively], with costs lower for NGS in 7/10 centers. As per the real‐world model, mean per‐biomarker NGS costs decreased over time (€231, €155, and €75 in the SP, CP, and FH scenarios, respectively) (Table 1B).

#### Impact of retesting on per‐patient costs in the real‐world model

In the real‐world model, the proportion of total NGS and SGT per‐patient costs attributable to retesting (confirmatory or indeterminate) varied across centers and scenarios (Table 2A). The mean proportion of per‐patient NGS and SGT costs attributable to retesting was less than 10% across centers in each temporal scenario.

**Table 2:** Impact of retesting in the real‐world model on (A) SGT and NGS costs per patient, and (B) differences in per‐patient costs for NGS versus SGT

(A)																	
			SP						CP						FH		
			SGT per‐patient costs			NGS per‐patient costs			SGT per‐patient costs			NGS per‐patient costs			NGS per‐patient costs		
Center	Number of patients tested during 1 January–31 December 2021	Number of years of experience using NGS	With retests, €	No retests, €	% of retest costs	With retests, €	No retests, €	% of retest costs	With retests, €	No retests, €	% of retest costs	With retests, €	No retests, €	% of retest costs	With retests, €	No retests, €	% of retest costs
1	832	9	3,772	3,772	0	1,972	1,890	4	5,223	5,223	0	1,504	1,282	15	3,277	3,173	3
2	341	12	1,786	1,776	1	2,087	1,722	17	1,786	1,776	1	2,087	1,722	17	1,484	1,108	25
3	314	5	2,817	2,275	19	857	728	15	2,817	2,275	19	1,159	1,030	11	1,412	1,283	9
4	195	11	2,353	2,160	8	1,664	1,478	11	2,353	2,160	8	2,054	1,834	11	3,010	2,617	13
5	811	>6	2,812	2,679	5	2,633	2,370	10	3,099	2,965	4	2,624	2,361	10	3,192	2,929	8
6	580	8*	766	622	19	876	802	8	1,564	1,177	25	1,459	1,360	7	1,612	1,513	6
7	181	10^†^	380	345	9	517	517	0	754	720	5	406	406	0	406	406	0
8	789	7	3,280	3,262	1	1,478	1,449	2	5,623	5,606	0	3,112	3,073	1	1,096	1,040	5
9	213	NA	2,733	2,632	4	3,399	3,118	8	3,142	3,040	3	3,788	3,559	6	3,844	3,619	6
10	235	8	982	982	0	2,215	2,215	0	1,290	1,290	0	2,244	2,244	0	1,691	1,691	0
Mean	NC	NC	2,168	2,051	6	1,770	1,629	8	2,765	2,623	7	2,044	1,887	8	2,102	1,938	8

(B)								
Center	Number of patients tested during 1 January–31 December 2021	Number of years of experience using NGS	SP			CP		
			Δ NGS versus SGT when retesting was included, € (%)	Δ NGS versus SGT when retesting was not included, € (%)	Δ inclusion versus exclusion of retesting, € (%)	Δ € NGS versus SGT when retesting was included, € (%)	Δ NGS versus SGT when retesting was not included, € (%)	Δ inclusion versus exclusion of retesting, € (%)
1	832	9	−1,800 (−48)	−1,881 (−50)	81 (2)	−3,719 (−71)	−3,941 (−75)	222 (4)
2	341	12	301 (17)	−55 (−3)	356 (20)	301 (17)	−55 (−3)	356 (20)
3	314	5	−1,960 (−70)	−1,547 (−68)	−413 (−2)	−1,658 (−59)	−1,246 (−55)	−412 (−4)
4	195	11	−689 (−29)	−682 (−32)	−7 (3)	−298 (−13)	−325 (−15)	27 (2)
5	811	>6	−179 (−6)	−308 (−12)	129 (6)	−475 (−15)	−604 (−20)	129 (5)
6	580	8*	110 (14)	180 (29)	−70 (−15)	−105 (−7)	183 (16)	−288 (−23)
7	181	10^†^	137 (36)	172 (50)	−35 (−14)	−348 (−46)	−314 (−44)	−34 (−2)
8	789	7	−1,802 (−55)	−1,813 (−56)	11 (1)	−2,511 (−45)	−2,533 (−45)	22 (0)
9	213	NA	666 (24)	486 (18)	180 (6)	646 (21)	518 (17)	128 (4)
10	235	8	1,233 (126)	1,233 (125)	0 (0)	954 (74)	954 (74)	0 (0)
Mean	NC	NC	−398 (−18)	−422 (−21)	24 (3)	−721 (−26)	−736 (−28)	15 (2)

Green cells represent lower costs for NGS versus SGT; orange cells represent higher costs for NGS versus SGT. Retests included both confirmatory tests and repeat tests owing to indeterminate results.

CP, Current Practice; FH, Future Horizons; NA, not available; NC, not calculated; NGS, next‐generation sequencing; SGT, single‐gene testing; SP, Starting Point.

* Center 6 used NGS partially since 2015.

^†^ Center 7 used NGS for predictive testing (for all cancer types) since 2002, as well as for diagnostic testing since 2013.

In the SP and CP scenarios, the mean center difference in per‐patient costs for NGS versus SGT was similar whether retesting was included in cost calculations or not (SP: 18% versus 21% lower for NGS than SGT, respectively; CP: 26% versus 28% lower for NGS than SGT, respectively) (Table 2B). Inclusion of retesting in cost calculations did not affect whether NGS or SGT was the more expensive testing strategy in all centers, with the exceptions of center 2 in the SP scenario, and centers 2 and 6 in the CP scenario.

#### Deterministic sensitivity analysis

In the DSA, the impact of altering cost input variables by ±20% on the total annual costs of NGS versus SGT was determined for each center in the SP and CP scenarios; the DSA was not conducted for the FH scenario, given that SGT costs were not available. For all centers in each scenario and in both models, consumable costs had the greatest impact on the difference in annual total testing costs of SGT and NGS (supplementary material, Figure S5).

In both models, increasing or decreasing the cost of the most impactful variable by 20% in the SP and CP scenarios did not affect whether SGT and NGS was associated with higher annual costs in any of the centers, with the exception of center 5 in the SP scenario of the real‐world model (supplementary material, Table S6A) and center 6 in the CP scenario of the standardized model (supplementary material, Table S6B).

## Discussion

This global study provides real‐world insights into the costs of biomarker testing using SGT and NGS over time in 4,491 patients with nonsquamous aNSCLC. We employed a micro‐costing approach to evaluate testing costs in 10 centers across three temporal scenarios (SP, CP, and FH) using two models: a real‐world model and a standardized model that included fixed sets of biomarkers, enabling inter‐site comparisons of costs. Across centers, and in both models, per‐patient and per‐biomarker costs of NGS decreased relative to those of SGT in the CP versus SP scenario, with reductions in per‐biomarker NGS costs seen over time. The minimum number of biomarkers required for NGS to result in cost savings versus SGT at the patient level was, on average, 10 in the SP scenario and 12 in the CP scenario, although this number varied across centers. Results were consistent, regardless of whether retesting was included in cost calculations, and were robust to variation in cost parameters.

In the SP scenario, NGS was associated with lower per‐patient costs compared with SGT in the real‐world model, and higher per‐patient costs in the standardized model. However, NGS was associated with lower per‐patient costs compared with SGT in the CP scenario in both models. This was expected, given the greater number of biomarkers tested and thus assays required for SGT in the later scenario. Accordingly, the number of SGT runs per patient was higher for most centers in the CP scenario of both models. Congruent with these findings, early cost comparison studies reported higher absolute diagnostic costs with NGS versus SGT [30, 31], but recent studies based on larger sets of biomarkers have demonstrated cost savings with NGS [24, 26, 27, 28, 29, 32]. For example, a 2023 study employing a genomic testing cost calculator based on biomarkers with approved targeted therapies demonstrated a reduced cost for NGS (€658) versus SGT (€1,983) per each correctly identified patient with nonsquamous aNSCLC [27]. A more recent population‐based study of public reimbursement data for aNSCLC in British Columbia, Canada, showed that multigene panel NGS testing has a moderate‐to‐high probability of increased cost‐effectiveness versus SGT at higher willingness‐to‐pay thresholds [29]. In our study, per‐biomarker costs were substantially lower for NGS than for SGT for most centers in both the SP and CP scenarios in the real‐world model. In the standardized model, the higher per‐biomarker costs of NGS in the SP scenario may have been due to the relatively low number of biomarkers compared with the real‐world model: 7 versus 14, respectively.

In both models, per‐patient NGS costs were slightly higher in the FH scenario compared with the CP scenario, likely due to the greater numbers of biomarkers tested and, therefore, the larger, more costly panels required. However, at the per‐biomarker level, NGS was substantially less expensive in the FH scenario compared with the CP scenario. This was expected, given that, in the FH versus earlier scenarios, most centers tested a greater number of biomarkers using larger NGS panels requiring fewer or a similar number of machine runs. Interestingly, only one center (center 5) reported using whole‐exome sequencing (WES)/whole‐genome sequencing (WGS) in the FH scenario. Use of WES/WGS may become more common over time, provided costs decrease and the technique is shown to identify more patients with actionable targets [33, 34, 35].

High variation in per‐patient costs was observed across centers for each technique in both models. As local pricing was used, this was likely due to differences between the countries included in our study. In line with other studies [36, 37, 38], testing costs were largely driven by consumables. Although the costs of some consumables, such as reagents, were likely comparable between centers, prices may have differed based on volume and/or whether other departments within the same institution used the same testing kits/components. Equipment and maintenance costs also differed substantially between sites, with some reporting negligible costs owing to equipment being included as part of consumable supply agreements, and other reporting costs exceeding 40% of the total testing costs per patient. Retests (both confirmatory and indeterminate) accounted for a low proportion of costs and appeared unrelated to total per‐patient costs; they also did not affect whether SGT or NGS was the less‐expensive testing technique in most centers.

The tipping point of 12 biomarkers for NGS, resulting in lower per‐patient costs compared with SGT in the CP scenario, was very similar to the number of biomarkers recommended for testing by the 2023 ESMO guidelines (11) and NCCN Guidelines® (10; both inclusive of PD‐L1) [6, 7, 8]. Some single‐center studies have reported cost savings for NGS over SGT when testing as few as five or six biomarkers [25, 39]. Indeed, the number of biomarkers needed for NGS to result in cost savings varied across centers in our study, ranging from 5 to 15 in the SP scenario, and from 4 to 18 in the CP scenario. The number of targetable biomarkers is likely to increase in the coming years as a result of new drug development [5, 40, 41, 42]. In addition, prognostic and predictive co‐mutations (e.g., *TP53*, *STK11*, *KEAP1*) [43], as well as predictive biomarkers that require broad sequence coverage (e.g., tumor mutational burden), are further increasing the complexity of molecular diagnosis in aNSCLC. As the number of biomarkers recommended for molecular testing in aNSCLC increases, improving testing efficiency will be key for payers and patients.

Though upfront costs of NGS remain high, NGS devices should enable long‐term scalability as the biomarker landscape evolves, with costs expected to decrease over time as more centers adopt the strategy and the technology improves. In laboratories that test multiple conditions, large, comprehensive NGS panels should improve cost efficiencies by increasing the volume of samples that can be tested using the same system, and by optimizing the workflow. Centralization of molecular testing may help further control costs and aid reimbursement [40, 44]. Looking ahead, it will be important to consider the cost implications of SGT and NGS beyond those of testing alone, such as the costs of treatments themselves. For example, compared with patients tested with NGS, those tested with SGT may be more likely to receive suboptimal nontargeted treatment due to undetected genomic alterations [10, 11, 13, 23, 24, 45, 46], which in turn may increase overall costs of treatment compared with upfront targeted therapy [10].

Our study had several limitations. For example, overhead costs were not collected for each center, but a fixed rate of 20% of the total testing costs was applied across sites. However, in the case where overhead costs had been collected, an accurate comparison of costs for each center/technique would have not been possible due to variations in the different allocation approaches used (e.g., the level of data aggregation, cost items included, and allocation drivers). The biomarkers included in the FH scenario were based on those expected to be recommended for testing in 3–4 years' time, and thus are hypothetical. Reimbursement policies may have affected the number of biomarkers considered/tested by each center in the real‐world model. Reimbursement of NGS in aNSCLC varies significantly across regulatory bodies and between countries in Asia and Europe, as well as between the public and private sectors [16, 17, 18, 47]; however, the impact of reimbursement on testing and costs was beyond the scope of this analysis. The standardized model included a fixed set of biomarkers tested across centers for each scenario, with center‐specific costs. However, some centers may have used larger NGS panels appropriate for testing higher numbers of biomarkers than were included in the model, potentially resulting in skewed NGS costs. In the analysis of the number of biomarkers required for NGS to result in cost savings over SGT, it was assumed that per‐patient SGT costs increased linearly with the number of biomarkers tested, but costs will likely have varied depending on the biomarker and SGT technique used. Lastly, neither the impact of inflation on costs over time nor differences in individual countries' gross domestic products and other country‐specific costs was considered.

This study focused on evaluating and comparing the costs of NGS and SGT in nonsquamous aNSCLC, based on real‐world data. Future studies are needed to examine the cost‐effectiveness of different testing strategies, taking into account the epidemiological distribution of biomarkers, choice of targeted treatment, and patient outcomes. For example, the broader cost‐effectiveness of testing should be considered within the context of subsequent downstream costs of biologic treatments, which can become prohibitive in the case of treatment with multiple consecutive biologics during advanced disease stages. Finally, reimbursement of testing costs is a major factor that will govern future integration of NGS into the clinic. A recent expert review focusing on economic evaluations of NGS in oncology highlighted the current need for high‐quality real‐world evidence in this field, which is vital for informing regulatory and reimbursement decisions for NGS. The findings from our study add to the body of robust real‐world data supporting the cost‐effectiveness of NGS compared with SGT [22].

In conclusion, this study provides global real‐world evidence for cost savings with NGS compared with SGT in current and future clinical practice scenarios for patients with aNSCLC. In the increasingly complex genomic landscape of lung cancer, widespread adoption of NGS, together with further improvements in the technology, may result in a more efficient use of limited healthcare resources.

## Author contributions statement

JK, VLRA and AB conceived this study. JK acquired the funding for and supervised the study. UM, C‐CC, EdÁ, PH, DK, T‐JK, TKHL, AR, AAS, ES, GT, MB, Y‐LC, GST, CHM, MM, AtE, HV and AS were involved in the study investigations. JMF, VLRA and AB were involved in the data curation, formal analysis, methodology and data visualization. SG‐M, AG, JMF, VLRA and AB were involved in data validation. JK, SG‐M and AG were involved in the study administration. All authors were involved in writing the paper and approved the submitted and published versions.

References 48 and 49 are cited only in the supplementary material.

## Supporting information


File S1. Center questionnaire

Supplementary materials and methods
**Figure S1.** Mean number of machine runs per patient for SGT and NGS in the real‐world model
**Figure S2.** Mean number of machine runs per patient for SGT and NGS in the standardized model
**Figure S3.** Mean cost per biomarker for SGT and NGS in the real‐world model
**Figure S4.** Mean cost per biomarker for SGT and NGS in the standardized model
**Figure S5.** Deterministic sensitivity analysis of total annual testing cost differences for NGS versus SGT
**Table S1.** Abbreviations
**Table S2.** Biomarkers tested in the real‐world model
**Table S3.** SGT techniques used in the real‐world model
**Table S4.** NGS panels used in the real‐world model
**Table S5.** Minimum number of biomarkers required for per‐patient cost savings for NGS versus SGT in the standardized model
**Table S6.** Effect of varying the most impactful cost parameter by ±20% on total annual testing cost differences for NGS versus SGT

## Data Availability

Qualified researchers may request data from Amgen. Complete details are available at: http://www.amgen.com/datasharing.
