# Supplementary material for: Costs of biomarker testing in advanced non‐small cell lung cancer: a global study comparing next‐generation sequencing and single‐gene testing
Source: J Pathol Clin Res. 2025 Mar 7;11(2):e70018. doi: 10.1002/2056-4538.70018 (PMC11886603; doi:10.1002/2056-4538.70018)
Supplement: Supplementary file 2 — Supplementary materials and methods Figure S1. Mean number of machine runs per patient for SGT and NGS in the real‐world model Figure S2. Mean number of machine runs per patient for SGT and NGS in the standardized model Figure S3. Mean cost per biomarker for SGT and NGS in the real‐world model Figure S4. Mean cost per biomarker for SGT and NGS in the standardized model Figure S5. Deterministic sensitivity analysis of total annual testing cost differences for NGS versus SGT Table S1. Abbreviations Table S2. Biomarkers tested in the real‐world model Table S3. SGT techniques used in the real‐world model Table S4. NGS panels used in the real‐world model Table S5. Minimum number of biomarkers required for per‐patient cost savings for NGS versus SGT in the standardized model Table S6. Effect of varying the most impactful cost parameter by ±20% on total annual testing cost differences for NGS versus SGT [file CJP2-11-e70018-s001.pdf]

# Costs of biomarker testing in advanced non-small cell lung cancer: a global study comparing next-generation sequencing and single-gene testing

U Malapelle *et al. J Pathol Clin Res* https://doi.org/10.1002/2056-4538.70018

**Supplementary materials and methods**

**Supplementary Figures S1–S5**

**Supplementary Tables S1–S6**

Reference numbers refer to the list in the main paper.

---

## Supplementary materials and methods

### Study design

In the real-world model, the SP scenario included biomarkers tested in routine clinical practice at each individual center at the time the study protocol was developed (2021–2022). The CP scenario included biomarkers tested in the SP scenario, and those that the individual centers routinely tested during the period 2023–2024. The FH scenario included biomarkers in the SP and CP scenarios, as well as those that each individual center expected to test routinely using NGS in the next 3–4 years (by 2025–2028).

In the standardized model, the biomarkers included in each scenario were validated using a consensus survey shared with the investigators at the participating centers before data collection. Briefly, the SP scenario included biomarkers recommended for testing by clinical guidelines and for which there was an EMA-approved targeted therapy before or at the time of development of the study protocol. As per the real-world model, the CP scenario additionally included biomarkers routinely tested by all centers from 2023–2024. The FH scenario also included those expected to be routinely tested using NGS by all centers from 2025–2028.

### Data collection

When data such as equipment costs were not available at the site level, average validated data from other centers were used. In the standardized model, missing consumables costs (35% of tests, for example, for biomarkers not tested by the center) were imputed using the average cost for the respective testing technique for the center (26% of tests) or, if the center did not utilize the technique, using the average cost of the biomarker and technique from a center in the same continent (9% of tests). In the latter case, the average equipment cost was also used, with personnel times based on those from the Predictive Molecular Pathology Laboratory, Department of Public Health, University of Naples Federico II (Naples, Italy).

All data were handled anonymously by the respective centers, and procedures were followed in compliance with the Declaration of Helsinki [48]. No patient-level data of a

personal or sensitive nature were included in this research, and therefore approvals from Institutional Review Boards or Independent Ethics Committees were not required for this study.

**Data analysis**

Total costs included personnel, consumables, equipment and overheads, and were calculated according to previously validated methods [26]. In brief, consumable costs included kit costs (panel and kit-sequencing reagents), non-kit costs (non-kit–based reagents and other consumables), and bioinformatic software costs, when applicable. Kit and software costs were based on the financial data of each laboratory, whereas non-kit costs were estimated by the centers. These estimates varied depending on differences in the procedures and assays used and the completeness of the listed costs. Equipment investment depreciation was set to 5 years, with an annual maintenance cost of 10% of the purchase cost. Acquisition costs were re-calculated according to the proportion of time dedicated to non-squamous aNSCLC. In some centers, equipment was included in kit costs due to commercial agreements or was not purchased directly by the laboratory. Equipment saturation was assumed to be 100% for SGT and specified by the centers for NGS. The hands-on time spent by healthcare professionals on testing activities for each technique was analyzed, with total personnel time varying depending on the fixed and variable time required for each activity and the equipment machine saturation. Personnel time was monetized using gross annual salaries for the professionals involved. Total costs were increased by 20% to account for overheads, using the approach reported by Schwarze *et al* [49].

Missing consumable and equipment costs, as well as personnel times, were imputed for some biomarkers/techniques and/or techniques in the standardized model using average cost data. All costs were inclusive of VAT. Costs reported in non-Euro currencies were converted to Euros using the 2022 European Central Bank average annual exchange rate.

## Supplementary figures

**Figure S1.** Mean number of machine runs per patient for SGT and NGS in the real-world model.

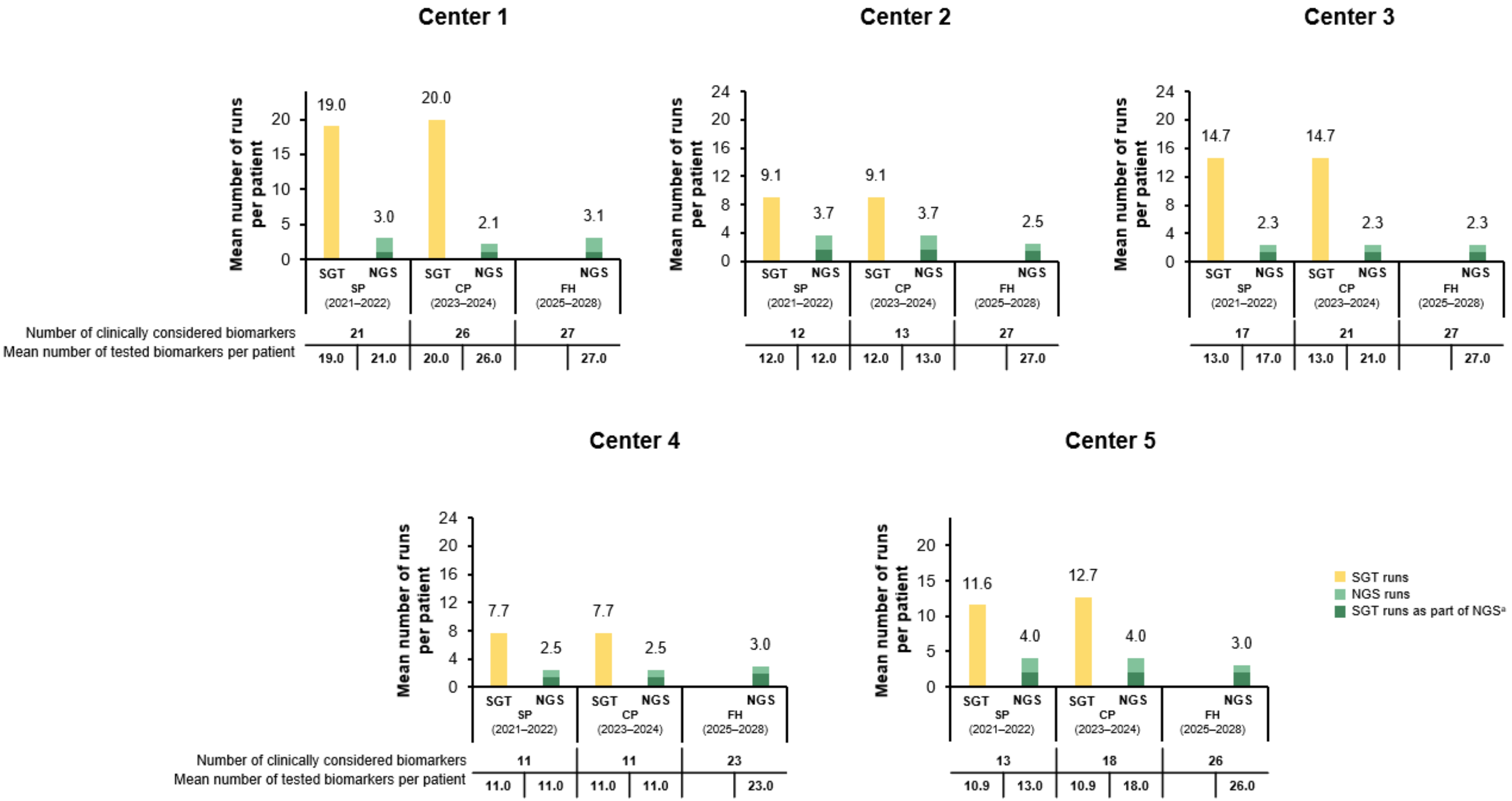

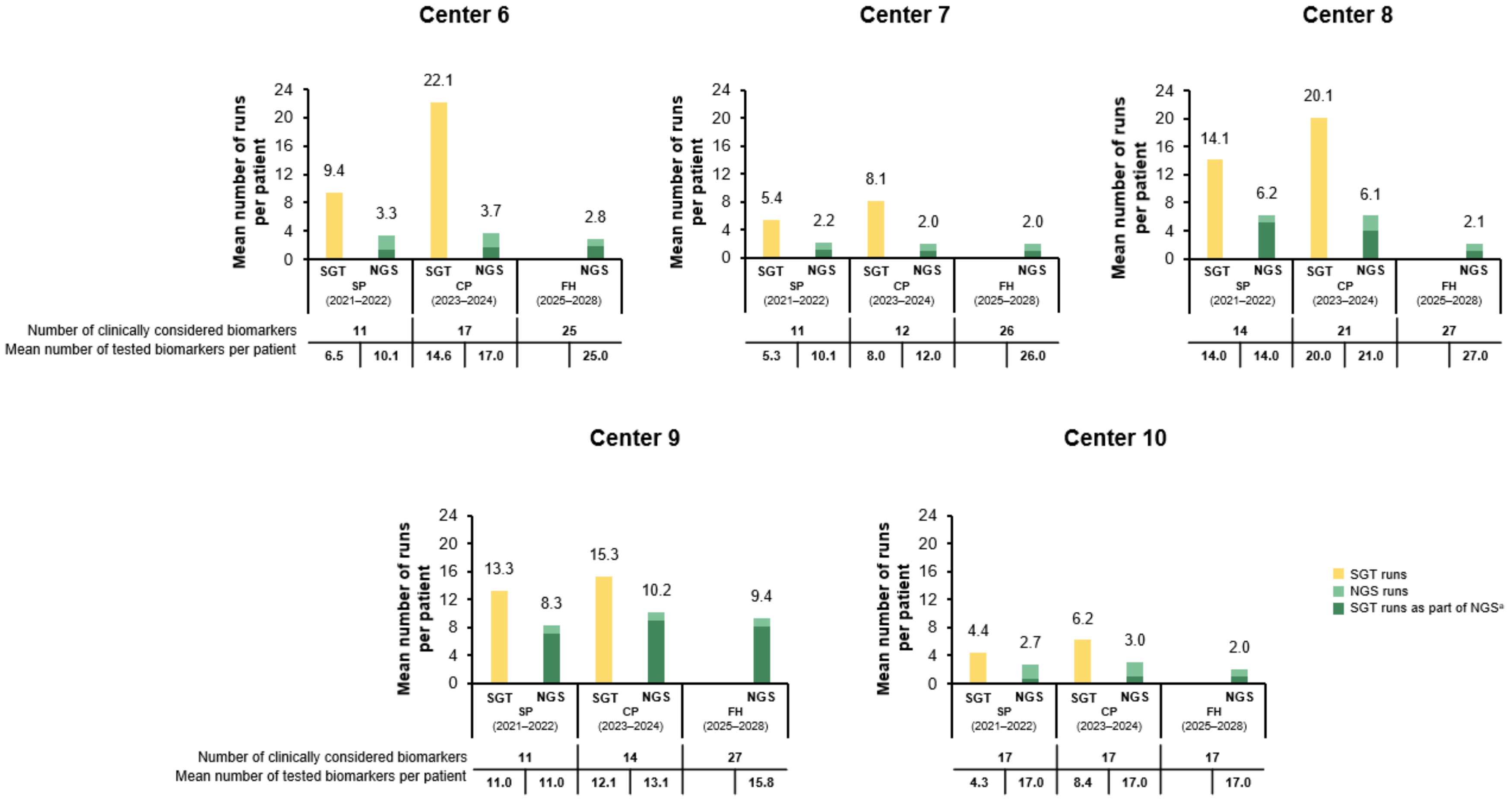


[a]For any additional biomarkers of interest not tested in NGS panels, including PD-L1 (in all scenarios).

Actual numbers of biomarkers tested may not have been integers in the case that they were tested in <100% of patients, or due to retesting.

CP, Current Practice; FH, Future Horizons; IHC, immunohistochemistry; NGS, next-generation sequencing; PD-L1, programmed cell death ligand 1; SGT, single gene testing; SP, Starting Point.

**Figure S2.** Mean number of machine runs per patient for SGT and NGS in the standardized model.

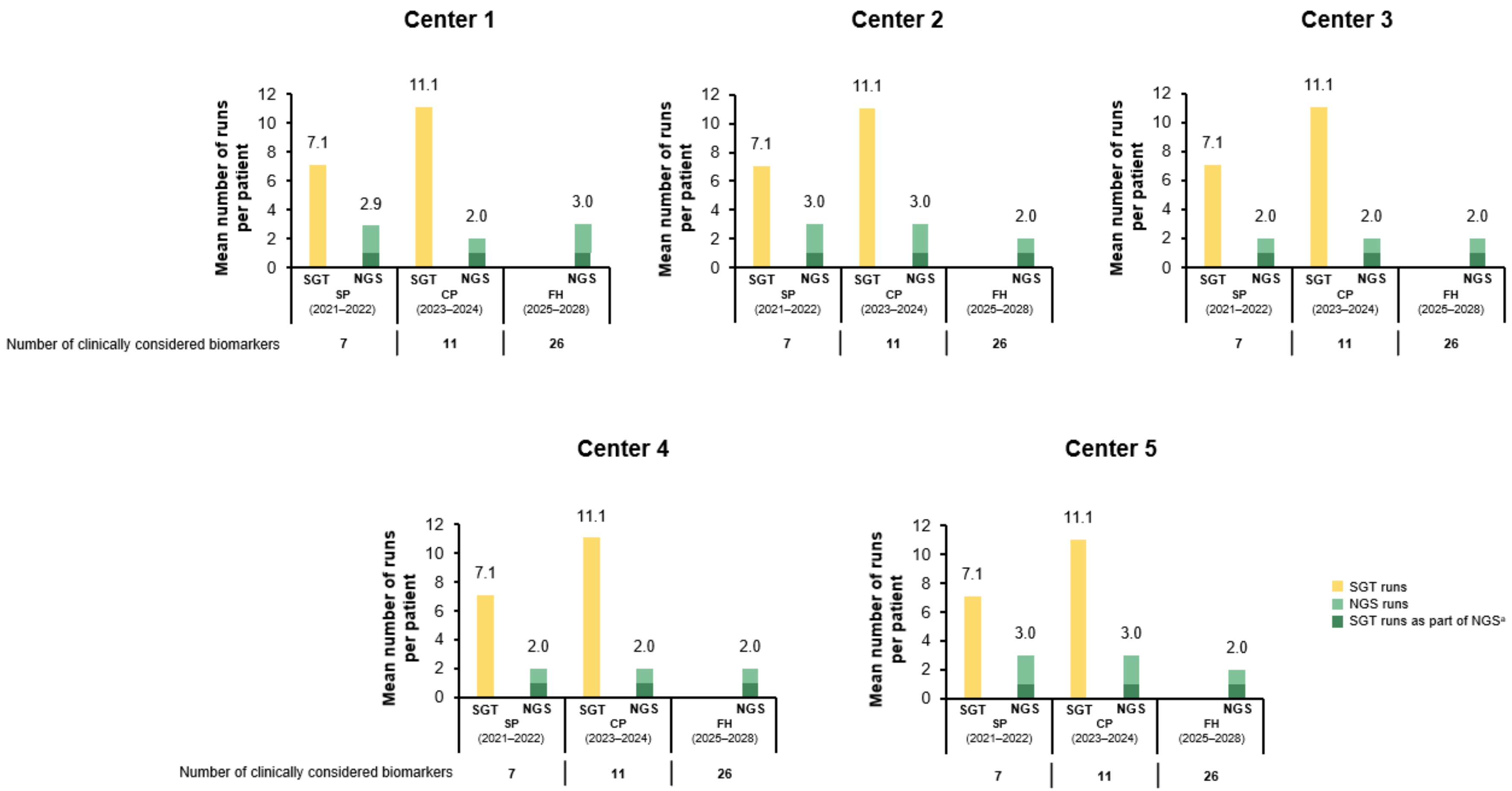

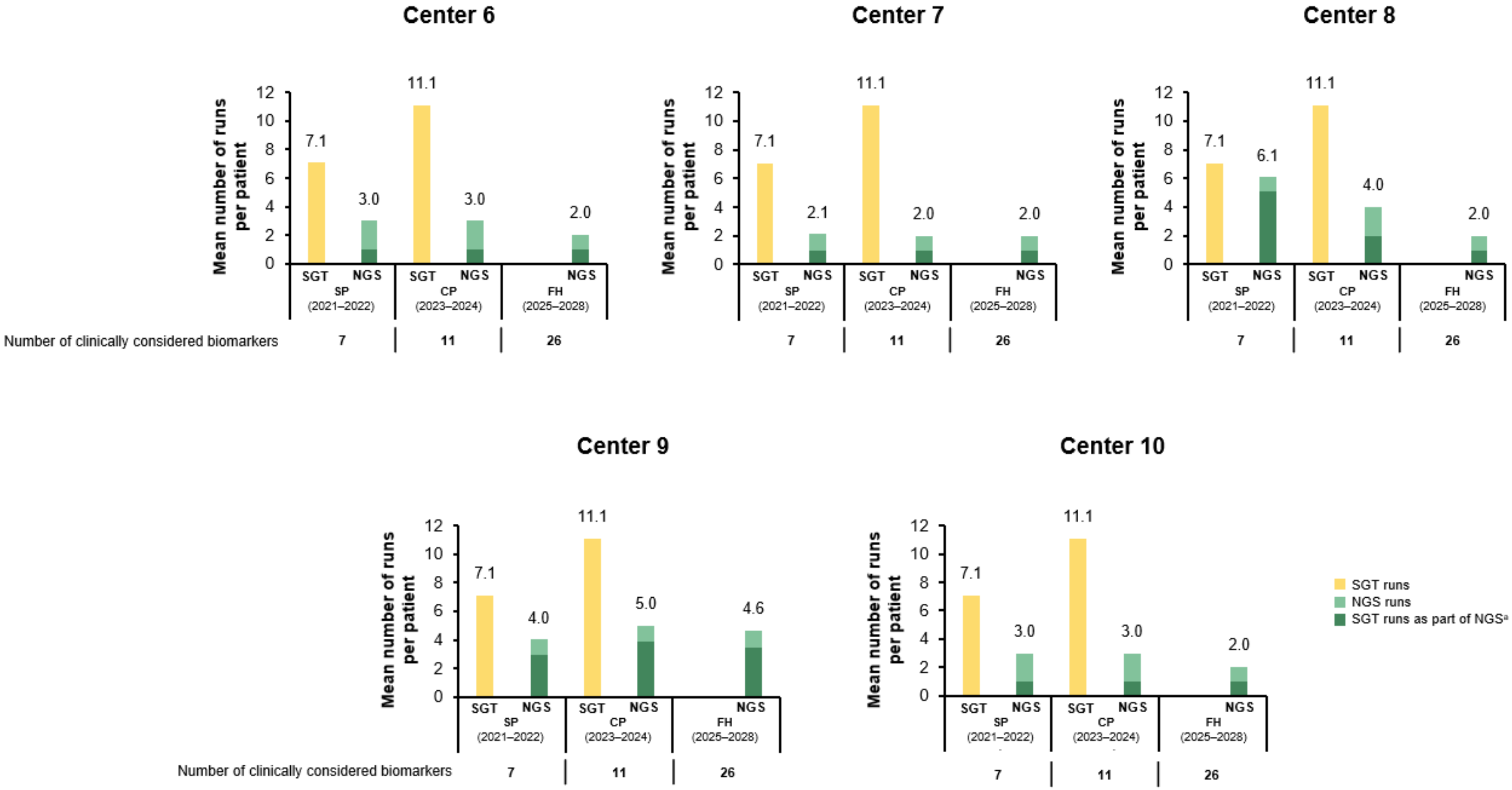


[a]For any additional biomarkers of interest not tested in NGS panels, including PD-L1 (in all scenarios).

Actual numbers of biomarkers tested may not have been integers in the case that they were tested in <100% of patients, or due to retesting.

CP, Current Practice; FH, Future Horizons; IHC, immunohistochemistry; NGS, next-generation sequencing; PD-L1, programmed cell death ligand 1; SGT, single gene testing; SP, Starting Point.

**Figure S3.** Mean cost per biomarker for SGT and NGS in the real-world model.

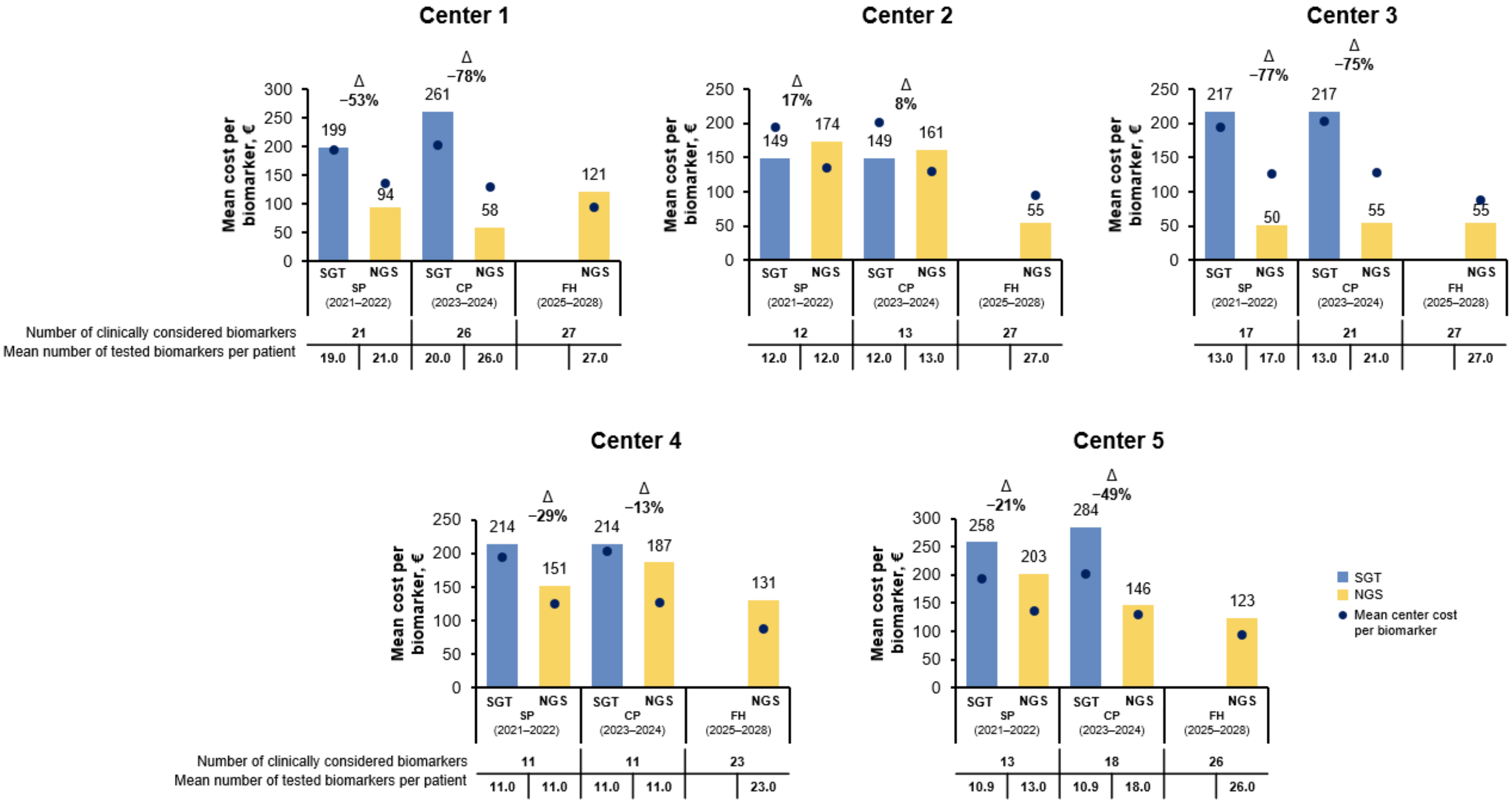

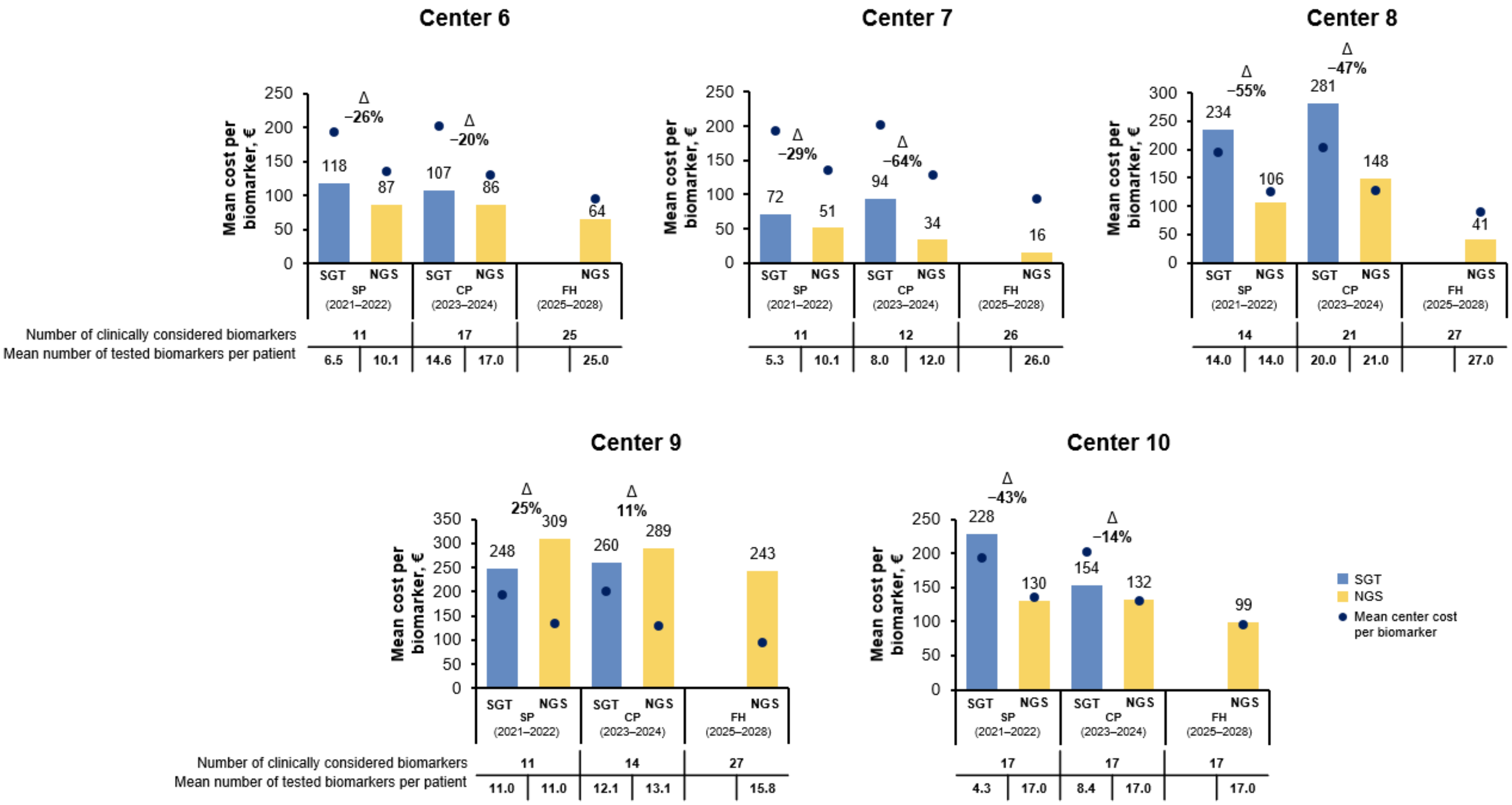


Actual numbers of biomarkers tested may not have been integers in the case that they were tested in <100% of patients, or due to retesting.

CP, Current Practice; FH, Future Horizons; NGS, next-generation sequencing; SGT, single-gene testing; SP, Starting Point.

**Figure S4.** Mean cost per biomarker for SGT and NGS in the standardized model.

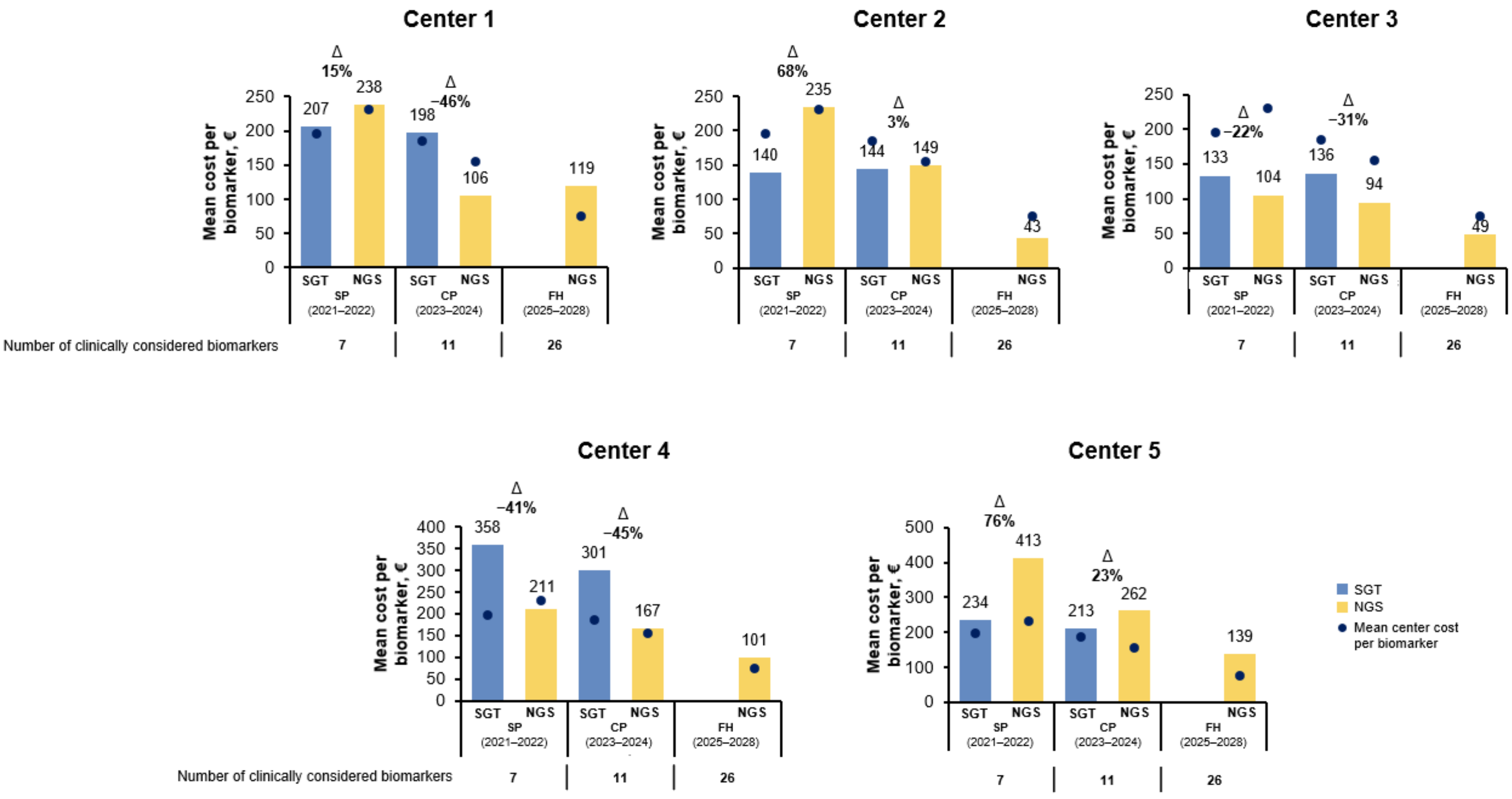

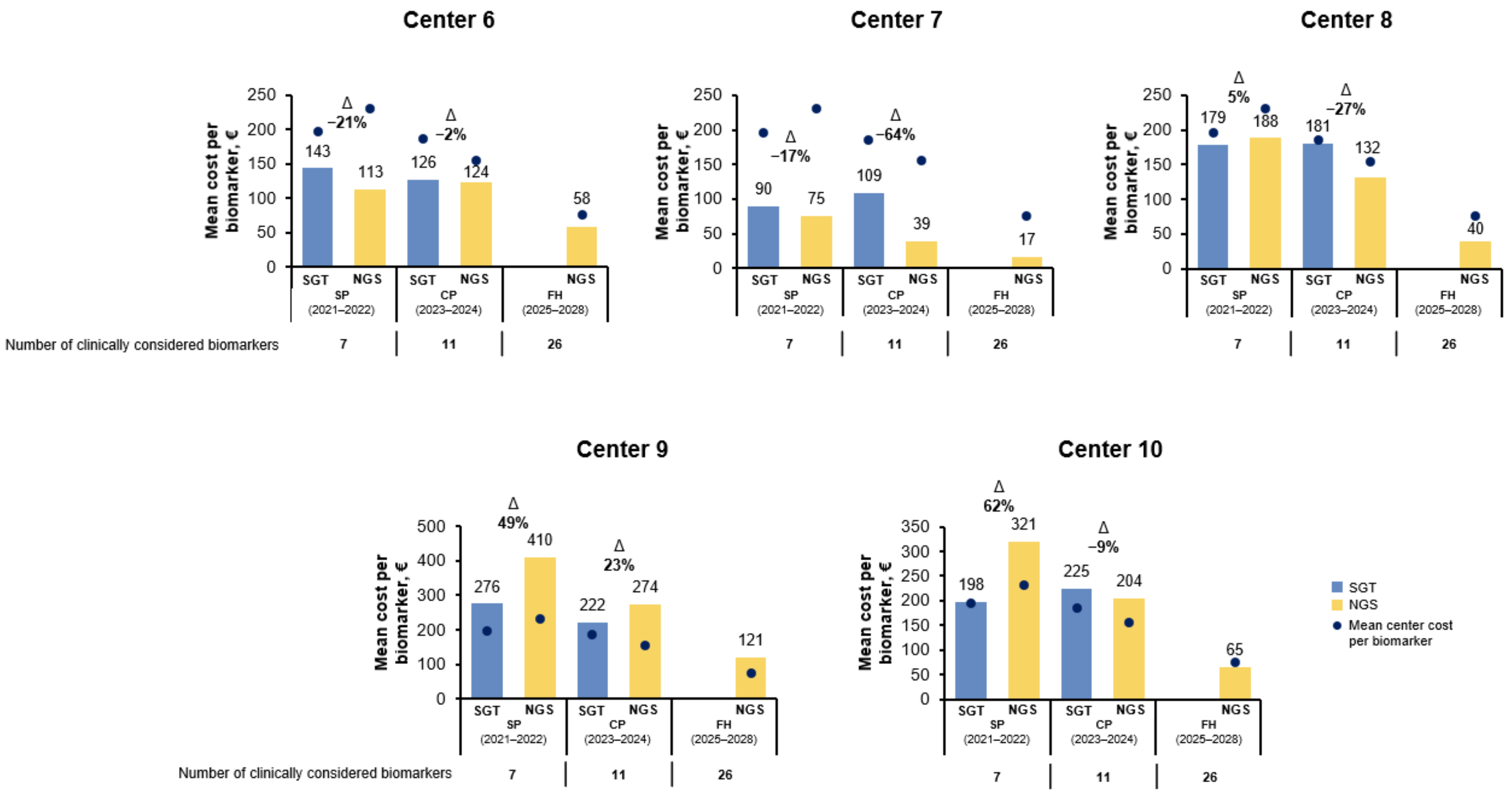


Actual numbers of biomarkers tested may not have been integers in the case that they were tested in <100% of patients, or due to retesting.

CP, Current Practice; FH, Future Horizons; NGS, next-generation sequencing; SGT, single-gene testing; SP, Starting Point.

**Figure S5.** Deterministic sensitivity analysis of total annual testing cost differences for NGS versus SGT, in the real-world model SP (A) and CP (B) scenarios, and in the standardized model SP (C) and CP (D) scenarios.

(A)

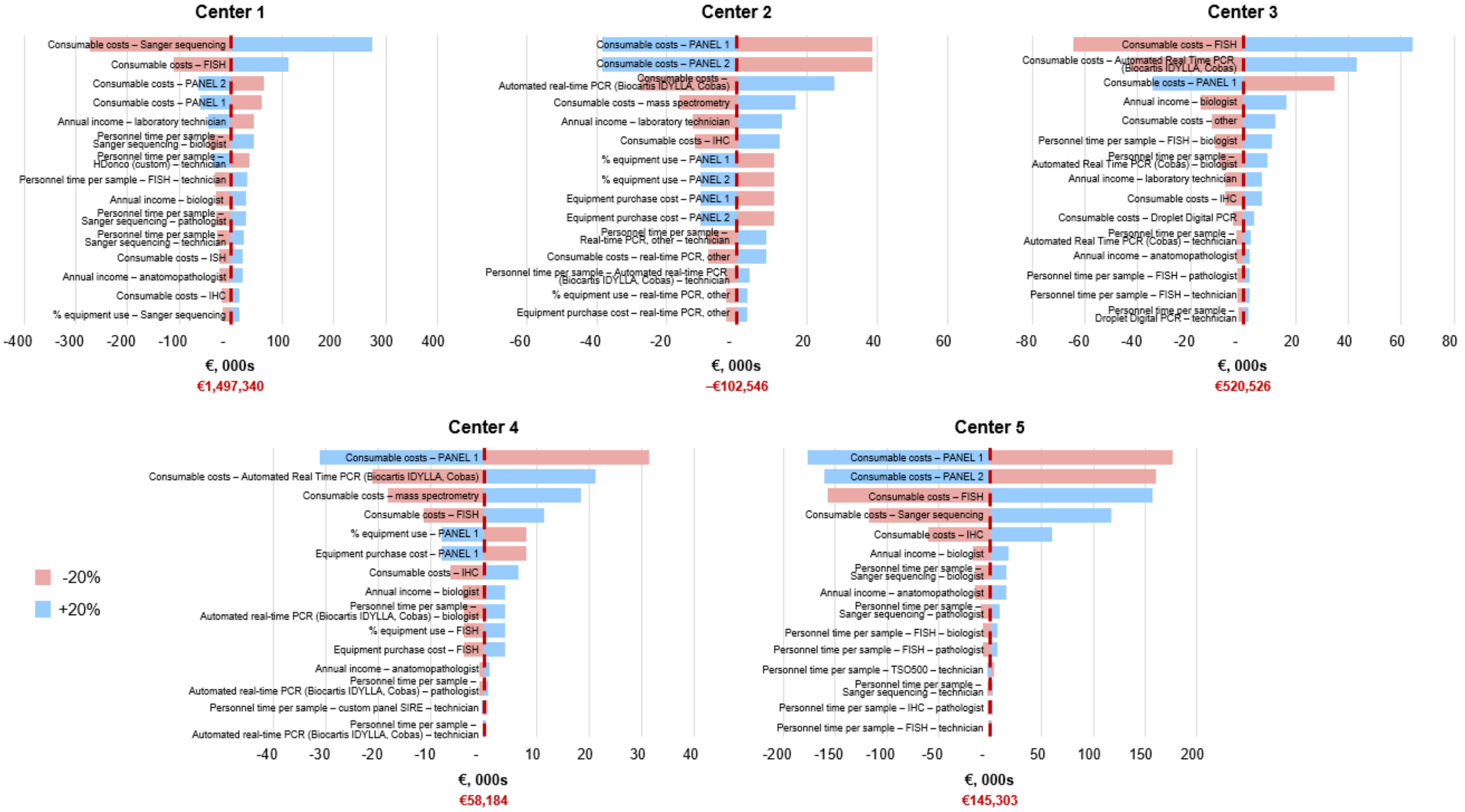

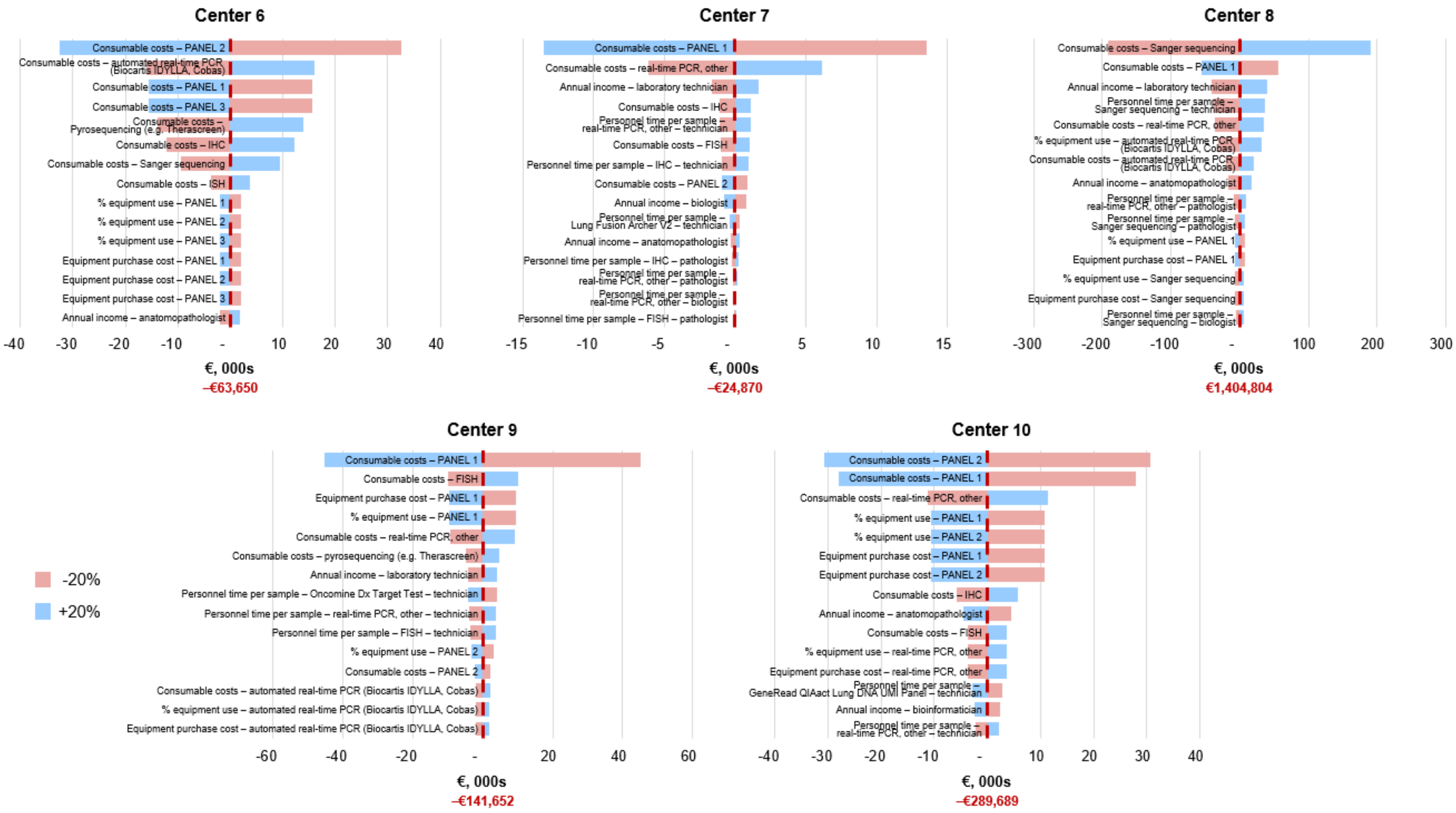
Center 6
Consumable costs – PANEL 2
Consumable costs – automated real-time PCR (Biocartis IDYLLA, Cobas)
Consumable costs – PANEL 1
Consumable costs – PANEL 3
Consumable costs – Pyrosequencing (e.g. Therascreen)
Consumable costs – IHC
Consumable costs – Sanger sequencing
Consumable costs – ISH
% equipment use – PANEL 1
% equipment use – PANEL 2
% equipment use – PANEL 3
Equipment purchase cost – PANEL 1
Equipment purchase cost – PANEL 2
Equipment purchase cost – PANEL 3
Annual income – anatomopathologist
-40 -30 -20 -10 - 10 20 30 40
€, 000s
–€63,650
Center 7
Consumable costs – PANEL 1
Consumable costs – real-time PCR, other
Annual income – laboratory technician
Consumable costs – IHC
Personnel time per sample – real-time PCR, other – technician
Consumable costs – FISH
Personnel time per sample – IHC – technician
Consumable costs – PANEL 2
Annual income – biologist
Personnel time per sample – Lung Fusion Archer V2 – technician
Annual income – anatomopathologist
Personnel time per sample – IHC – pathologist
Personnel time per sample – real-time PCR, other – pathologist
Personnel time per sample – real-time PCR, other – biologist
Personnel time per sample – FISH – pathologist
-15 -10 -5 - 5 10 15
€, 000s
–€24,870
Center 8
Consumable costs – Sanger sequencing
Consumable costs – PANEL 1
Annual income – laboratory technician
Personnel time per sample – Sanger sequencing – technician
Consumable costs – real-time PCR, other
% equipment use – automated real-time PCR (Biocartis IDYLLA, Cobas)
Consumable costs – automated real-time PCR (Biocartis IDYLLA, Cobas)
Annual income – anatomopathologist
Personnel time per sample – real-time PCR, other – pathologist
Personnel time per sample – Sanger sequencing – pathologist
% equipment use – PANEL 1
Equipment purchase cost – PANEL 1
% equipment use – Sanger sequencing
Equipment purchase cost – Sanger sequencing
Personnel time per sample – Sanger sequencing – biologist
-300 -200 -100 - 100 200 300
€, 000s
€1,404,804
-20%
+20%
Center 9
Consumable costs – PANEL 1
Consumable costs – FISH
Equipment purchase cost – PANEL 1
% equipment use – PANEL 1
Consumable costs – real-time PCR, other
Consumable costs – pyrosequencing (e.g. Therascreen)
Annual income – laboratory technician
Personnel time per sample – Oncomine Dx Target Test – technician
Personnel time per sample – real-time PCR, other – technician
Personnel time per sample – FISH – technician
% equipment use – PANEL 2
Consumable costs – PANEL 2
Consumable costs – automated real-time PCR (Biocartis IDYLLA, Cobas)
% equipment use – automated real-time PCR (Biocartis IDYLLA, Cobas)
Equipment purchase cost – automated real-time PCR (Biocartis IDYLLA, Cobas)
-60 -40 -20 - 20 40 60
€, 000s
–€141,652
Center 10
Consumable costs – PANEL 2
Consumable costs – PANEL 1
Consumable costs – real-time PCR, other
% equipment use – PANEL 1
% equipment use – PANEL 2
Equipment purchase cost – PANEL 1
Equipment purchase cost – PANEL 2
Consumable costs – IHC
Annual income – anatomopathologist
Consumable costs – FISH
% equipment use – real-time PCR, other
Equipment purchase cost – real-time PCR, other
Personnel time per sample – GeneRead QIAact Lung DNA UMI Panel – technician
Annual income – bioinformatician
Personnel time per sample – real-time PCR, other – technician
-40 -30 -20 -10 - 10 20 30 40
€, 000s
–€289,689

(B)

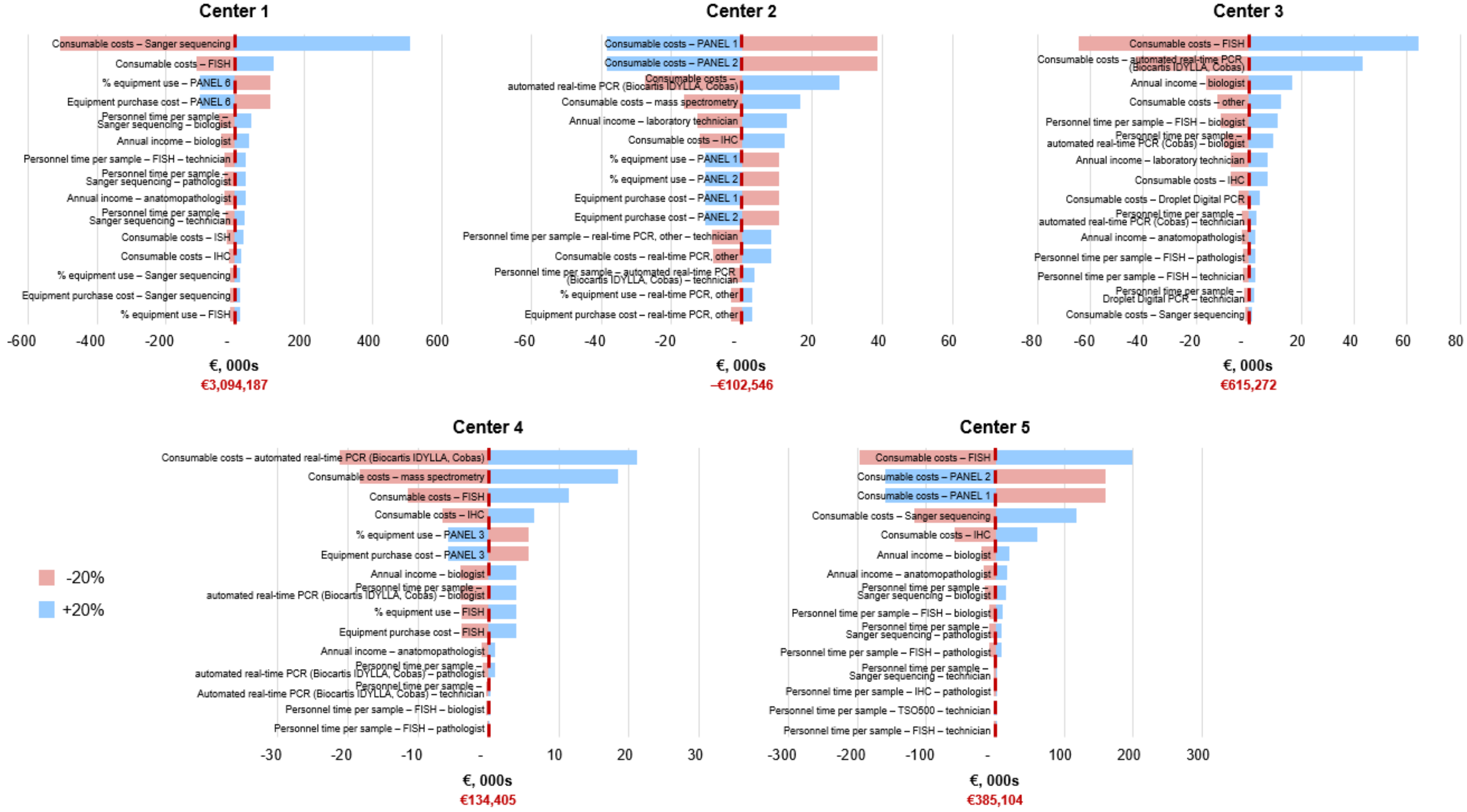

Center 1
Consumable costs – Sanger sequencing
Consumable costs – FISH
% equipment use – PANEL 6
Equipment purchase cost – PANEL 6
Personnel time per sample – Sanger sequencing – biologist
Annual income – biologist
Personnel time per sample – FISH – technician
Personnel time per sample – Sanger sequencing – pathologist
Annual income – anatomopathologist
Personnel time per sample – Sanger sequencing – technician
Consumable costs – ISH
Consumable costs – IHC
% equipment use – Sanger sequencing
Equipment purchase cost – Sanger sequencing
% equipment use – FISH
-600 -400 -200 - 200 400 600
€, 000s
€3,094,187
Center 2
Consumable costs – PANEL 1
Consumable costs – PANEL 2
Consumable costs – automated real-time PCR (Biocartis IDYLLA, Cobas)
Consumable costs – mass spectrometry
Annual income – laboratory technician
Consumable costs – IHC
% equipment use – PANEL 1
% equipment use – PANEL 2
Equipment purchase cost – PANEL 1
Equipment purchase cost – PANEL 2
Personnel time per sample – real-time PCR, other – technician
Consumable costs – real-time PCR, other
Personnel time per sample – automated real-time PCR (Biocartis IDYLLA, Cobas) – technician
% equipment use – real-time PCR, other
Equipment purchase cost – real-time PCR, other
-60 -40 -20 - 20 40 60
€, 000s
–€102,546
Center 3
Consumable costs – FISH
Consumable costs – automated real-time PCR (Biocartis IDYLLA, Cobas)
Annual income – biologist
Consumable costs – other
Personnel time per sample – FISH – biologist
Personnel time per sample – automated real-time PCR (Cobas) – biologist
Annual income – laboratory technician
Consumable costs – IHC
Consumable costs – Droplet Digital PCR
Personnel time per sample – automated real-time PCR (Cobas) – technician
Annual income – anatomopathologist
Personnel time per sample – FISH – pathologist
Personnel time per sample – FISH – technician
Personnel time per sample – Droplet Digital PCR – technician
Consumable costs – Sanger sequencing
-80 -60 -40 -20 - 20 40 60 80
€, 000s
€615,272
Center 4
Consumable costs – automated real-time PCR (Biocartis IDYLLA, Cobas)
Consumable costs – mass spectrometry
Consumable costs – FISH
Consumable costs – IHC
% equipment use – PANEL 3
Equipment purchase cost – PANEL 3
Annual income – biologist
Personnel time per sample – automated real-time PCR (Biocartis IDYLLA, Cobas) – biologist
% equipment use – FISH
Equipment purchase cost – FISH
Annual income – anatomopathologist
Personnel time per sample – automated real-time PCR (Biocartis IDYLLA, Cobas) – pathologist
Personnel time per sample – Automated real-time PCR (Biocartis IDYLLA, Cobas) – technician
Personnel time per sample – FISH – biologist
Personnel time per sample – FISH – pathologist
-30 -20 -10 - 10 20 30
€, 000s
€134,405
Center 5
Consumable costs – FISH
Consumable costs – PANEL 2
Consumable costs – PANEL 1
Consumable costs – Sanger sequencing
Consumable costs – IHC
Annual income – biologist
Annual income – anatomopathologist
Personnel time per sample – Sanger sequencing – biologist
Personnel time per sample – FISH – biologist
Personnel time per sample – Sanger sequencing – pathologist
Personnel time per sample – FISH – pathologist
Personnel time per sample – Sanger sequencing – technician
Personnel time per sample – IHC – pathologist
Personnel time per sample – TSO500 – technician
Personnel time per sample – FISH – technician
-300 -200 -100 - 100 200 300
€, 000s
€385,104
-20%
+20%

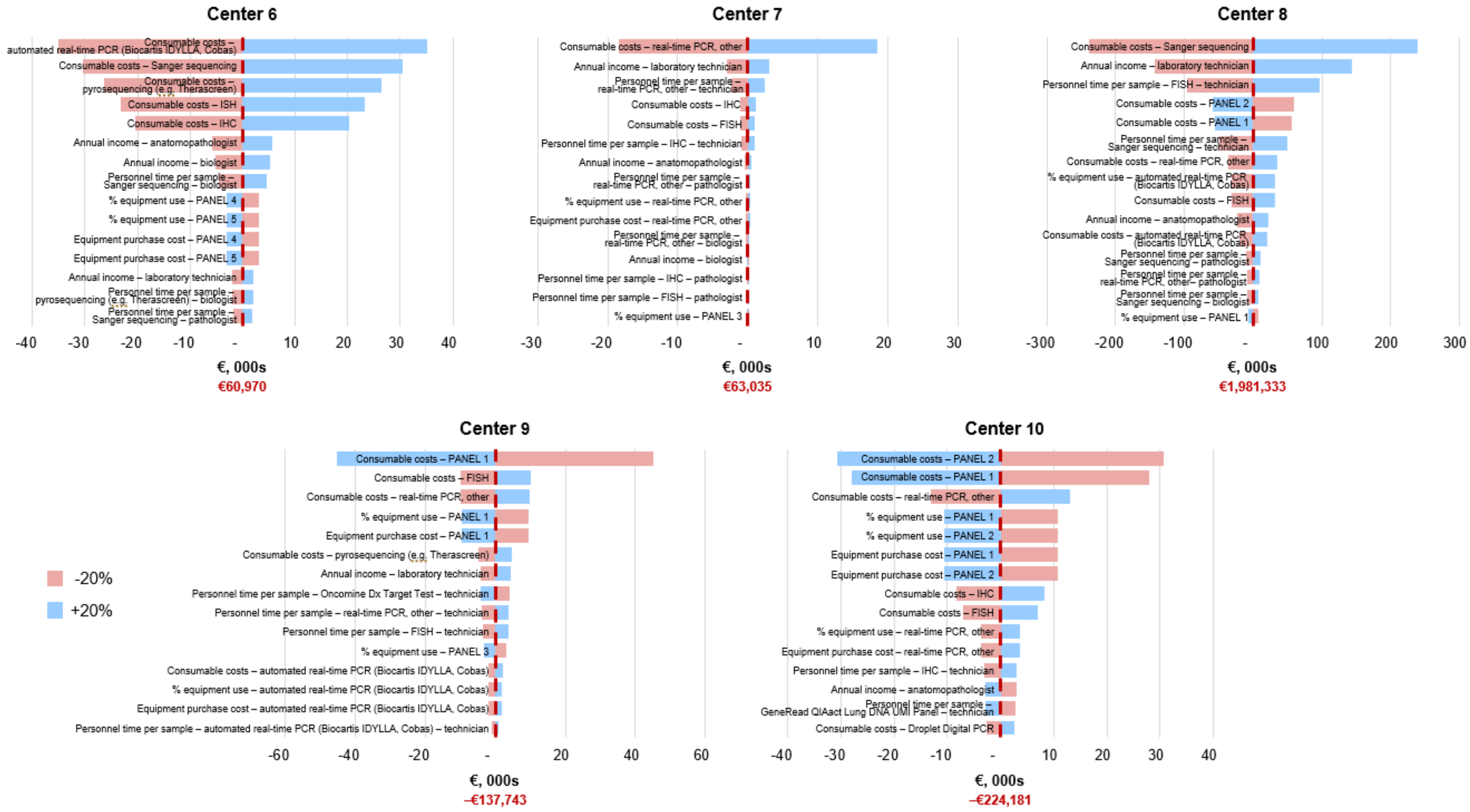
Center 6
€, 000s
€60,970
Center 7
€, 000s
€63,035
Center 8
€, 000s
€1,981,333
Center 9
€, 000s
–€137,743
Center 10
€, 000s
–€224,181
-20%
+20%

(C)

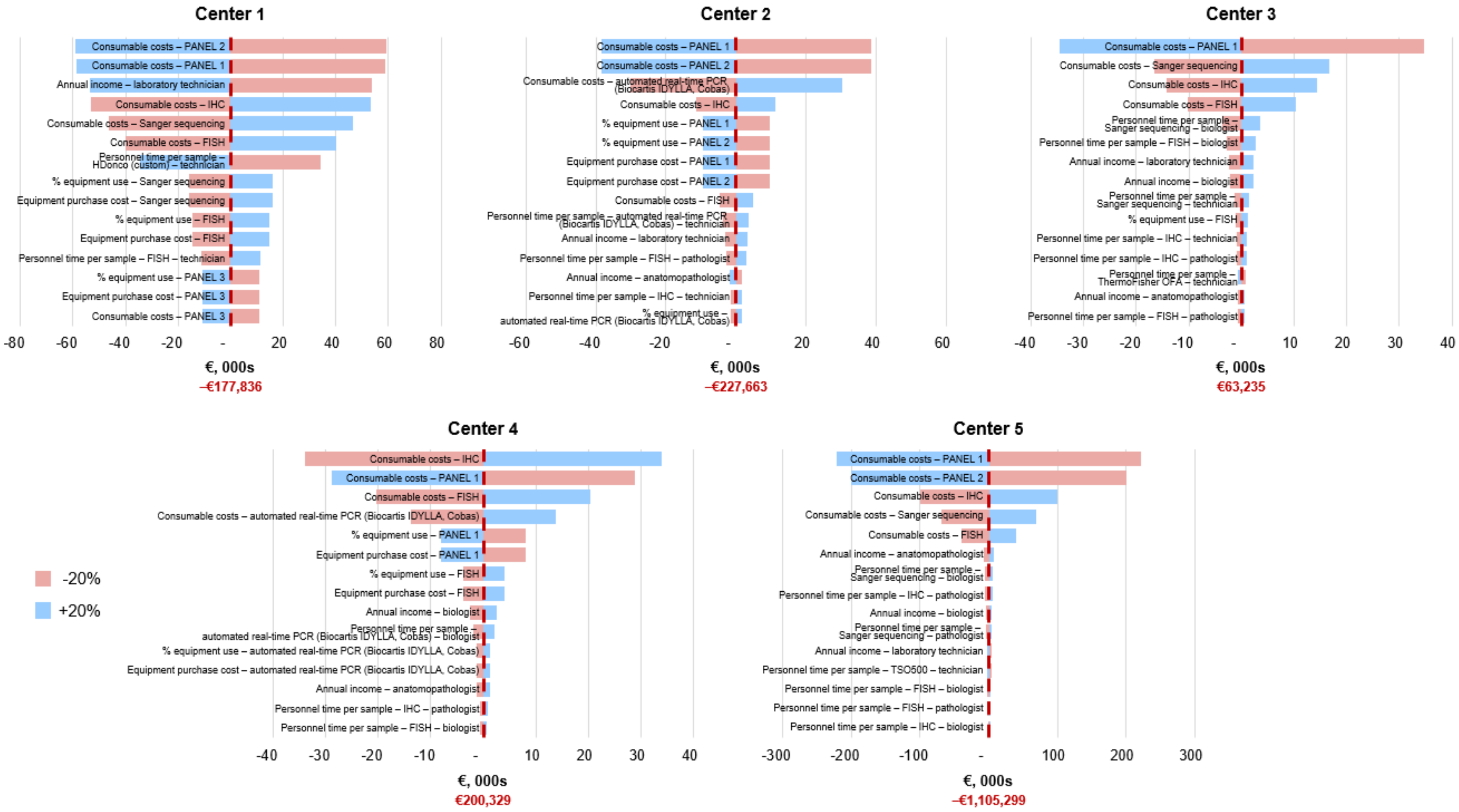
Center 1
Consumable costs – PANEL 2
Consumable costs – PANEL 1
Annual income – laboratory technician
Consumable costs – IHC
Consumable costs – Sanger sequencing
Consumable costs – FISH
Personnel time per sample – HDonco (custom) – technician
% equipment use – Sanger sequencing
Equipment purchase cost – Sanger sequencing
% equipment use – FISH
Equipment purchase cost – FISH
Personnel time per sample – FISH – technician
% equipment use – PANEL 3
Equipment purchase cost – PANEL 3
Consumable costs – PANEL 3
-80 -60 -40 -20 - 20 40 60 80
€, 000s
–€177,836
Center 2
Consumable costs – PANEL 1
Consumable costs – PANEL 2
Consumable costs – automated real-time PCR (Biocartis IDYLLA, Cobas)
Consumable costs – IHC
% equipment use – PANEL 1
% equipment use – PANEL 2
Equipment purchase cost – PANEL 1
Equipment purchase cost – PANEL 2
Consumable costs – FISH
Personnel time per sample – automated real-time PCR (Biocartis IDYLLA, Cobas) – technician
Annual income – laboratory technician
Personnel time per sample – FISH – pathologist
Annual income – anatomopathologist
Personnel time per sample – IHC – technician
% equipment use – automated real-time PCR (Biocartis IDYLLA, Cobas)
-60 -40 -20 - 20 40 60
€, 000s
–€227,663
Center 3
Consumable costs – PANEL 1
Consumable costs – Sanger sequencing
Consumable costs – IHC
Consumable costs – FISH
Personnel time per sample – Sanger sequencing – biologist
Personnel time per sample – FISH – biologist
Annual income – laboratory technician
Annual income – biologist
Personnel time per sample – Sanger sequencing – technician
% equipment use – FISH
Personnel time per sample – IHC – technician
Personnel time per sample – IHC – pathologist
Personnel time per sample – ThermoFisher OFA – technician
Annual income – anatomopathologist
Personnel time per sample – FISH – pathologist
-40 -30 -20 -10 - 10 20 30 40
€, 000s
€63,235
Center 4
Consumable costs – IHC
Consumable costs – PANEL 1
Consumable costs – FISH
Consumable costs – automated real-time PCR (Biocartis IDYLLA, Cobas)
% equipment use – PANEL 1
Equipment purchase cost – PANEL 1
% equipment use – FISH
Equipment purchase cost – FISH
Annual income – biologist
Personnel time per sample – automated real-time PCR (Biocartis IDYLLA, Cobas) – biologist
% equipment use – automated real-time PCR (Biocartis IDYLLA, Cobas)
Equipment purchase cost – automated real-time PCR (Biocartis IDYLLA, Cobas)
Annual income – anatomopathologist
Personnel time per sample – IHC – pathologist
Personnel time per sample – FISH – biologist
-40 -30 -20 -10 - 10 20 30 40
€, 000s
€200,329
Center 5
Consumable costs – PANEL 1
Consumable costs – PANEL 2
Consumable costs – IHC
Consumable costs – Sanger sequencing
Consumable costs – FISH
Annual income – anatomopathologist
Personnel time per sample – Sanger sequencing – biologist
Personnel time per sample – IHC – pathologist
Annual income – biologist
Personnel time per sample – Sanger sequencing – pathologist
Annual income – laboratory technician
Personnel time per sample – TSO500 – technician
Personnel time per sample – FISH – biologist
Personnel time per sample – FISH – pathologist
Personnel time per sample – IHC – biologist
-300 -200 -100 - 100 200 300
€, 000s
–€1,105,299
-20%
+20%

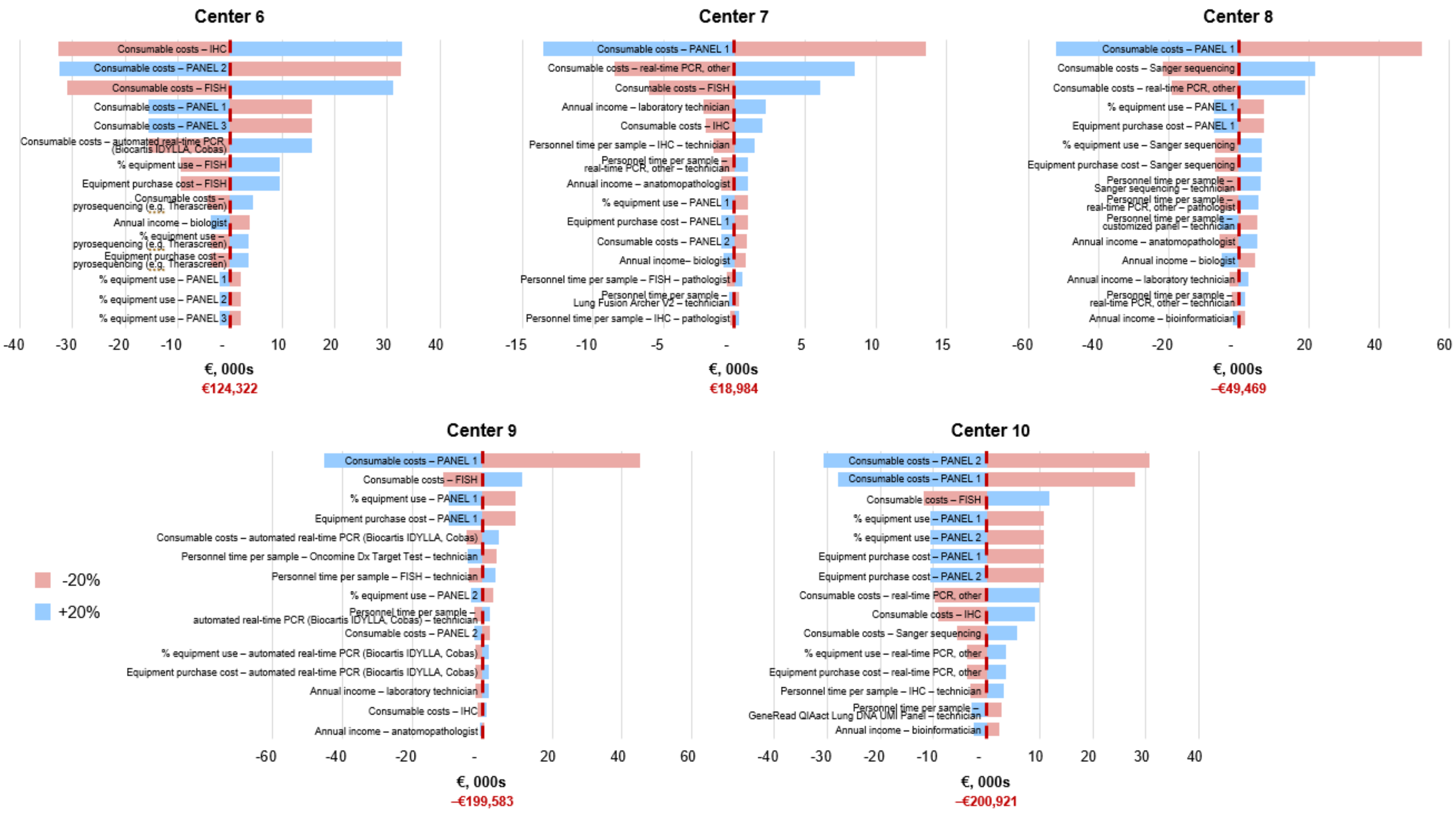
Center 6
Consumable costs – IHC
Consumable costs – PANEL 2
Consumable costs – FISH
Consumable costs – PANEL 1
Consumable costs – PANEL 3
Consumable costs – automated real-time PCR (Biocartis IDYLLA, Cobas)
% equipment use – FISH
Equipment purchase cost – FISH
Consumable costs – pyrosequencing (e.g. Therascreen)
Annual income – biologist
% equipment use – pyrosequencing (e.g. Therascreen)
Equipment purchase cost – pyrosequencing (e.g. Therascreen)
% equipment use – PANEL 1
% equipment use – PANEL 2
% equipment use – PANEL 3
-40 -30 -20 -10 - 10 20 30 40
€, 000s
€124,322
Center 7
Consumable costs – PANEL 1
Consumable costs – real-time PCR, other
Consumable costs – FISH
Annual income – laboratory technician
Consumable costs – IHC
Personnel time per sample – IHC – technician
Personnel time per sample – real-time PCR, other – technician
Annual income – anatomopathologist
% equipment use – PANEL 1
Equipment purchase cost – PANEL 1
Consumable costs – PANEL 2
Annual income– biologist
Personnel time per sample – FISH – pathologist
Personnel time per sample – Lung Fusion Archer V2 – technician
Personnel time per sample – IHC – pathologist
-15 -10 -5 - 5 10 15
€, 000s
€18,984
Center 8
Consumable costs – PANEL 1
Consumable costs – Sanger sequencing
Consumable costs – real-time PCR, other
% equipment use – PANEL 1
Equipment purchase cost – PANEL 1
% equipment use – Sanger sequencing
Equipment purchase cost – Sanger sequencing
Personnel time per sample – Sanger sequencing – technician
Personnel time per sample – real-time PCR, other – pathologist
Personnel time per sample – customized panel – technician
Annual income – anatomopathologist
Annual income – biologist
Annual income – laboratory technician
Personnel time per sample – real-time PCR, other – technician
Annual income – bioinformatician
-60 -40 -20 - 20 40 60
€, 000s
–€49,469
-20%
+20%
Center 9
Consumable costs – PANEL 1
Consumable costs – FISH
% equipment use – PANEL 1
Equipment purchase cost – PANEL 1
Consumable costs – automated real-time PCR (Biocartis IDYLLA, Cobas)
Personnel time per sample – Oncomine Dx Target Test – technician
Personnel time per sample – FISH – technician
% equipment use – PANEL 2
Personnel time per sample – automated real-time PCR (Biocartis IDYLLA, Cobas) – technician
Consumable costs – PANEL 2
% equipment use – automated real-time PCR (Biocartis IDYLLA, Cobas)
Equipment purchase cost – automated real-time PCR (Biocartis IDYLLA, Cobas)
Annual income – laboratory technician
Consumable costs – IHC
Annual income – anatomopathologist
-60 -40 -20 - 20 40 60
€, 000s
–€199,583
Center 10
Consumable costs – PANEL 2
Consumable costs – PANEL 1
Consumable costs – FISH
% equipment use – PANEL 1
% equipment use – PANEL 2
Equipment purchase cost – PANEL 1
Equipment purchase cost – PANEL 2
Consumable costs – real-time PCR, other
Consumable costs – IHC
Consumable costs – Sanger sequencing
% equipment use – real-time PCR, other
Equipment purchase cost – real-time PCR, other
Personnel time per sample – IHC – technician
Personnel time per sample – GeneRead QIAact Lung DNA UMI Panel – technician
Annual income – bioinformatician
-40 -30 -20 -10 - 10 20 30 40
€, 000s
–€200,921

(D)

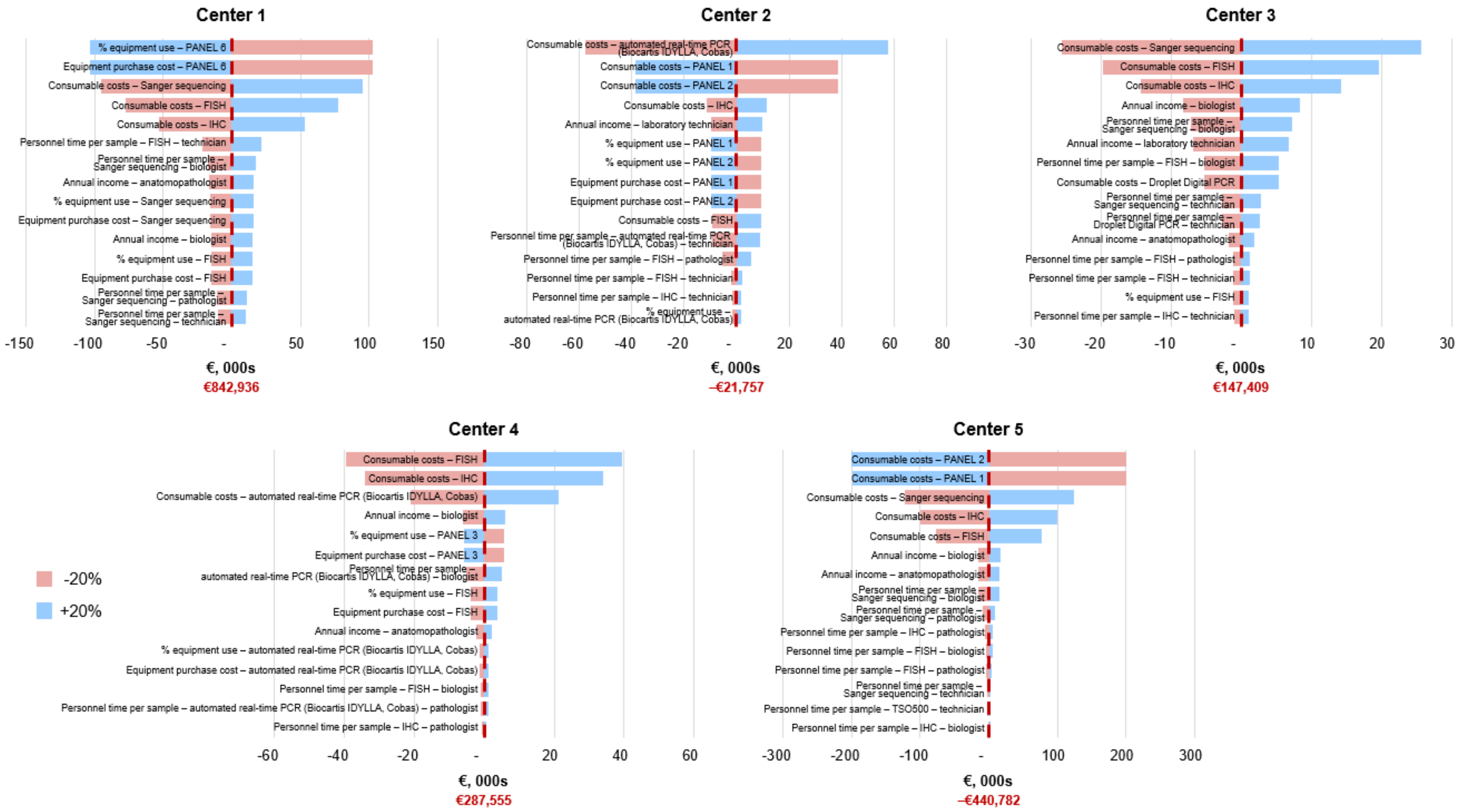

Center 1
% equipment use – PANEL 6
Equipment purchase cost – PANEL 6
Consumable costs – Sanger sequencing
Consumable costs – FISH
Consumable costs – IHC
Personnel time per sample – FISH – technician
Personnel time per sample – Sanger sequencing – biologist
Annual income – anatomopathologist
% equipment use – Sanger sequencing
Equipment purchase cost – Sanger sequencing
Annual income – biologist
% equipment use – FISH
Equipment purchase cost – FISH
Personnel time per sample – Sanger sequencing – pathologist
Personnel time per sample – Sanger sequencing – technician
-150
-100
-50
-
50
100
150
€, 000s
€842,936
Center 2
Consumable costs – automated real-time PCR (Biocartis IDYLLA, Cobas)
Consumable costs – PANEL 1
Consumable costs – PANEL 2
Consumable costs – IHC
Annual income – laboratory technician
% equipment use – PANEL 1
% equipment use – PANEL 2
Equipment purchase cost – PANEL 1
Equipment purchase cost – PANEL 2
Consumable costs – FISH
Personnel time per sample – automated real-time PCR (Biocartis IDYLLA, Cobas) – technician
Personnel time per sample – FISH – pathologist
Personnel time per sample – FISH – technician
Personnel time per sample – IHC – technician
% equipment use – automated real-time PCR (Biocartis IDYLLA, Cobas)
-80
-60
-40
-20
-
20
40
60
80
€, 000s
–€21,757
Center 3
Consumable costs – Sanger sequencing
Consumable costs – FISH
Consumable costs – IHC
Annual income – biologist
Personnel time per sample – Sanger sequencing – biologist
Annual income – laboratory technician
Personnel time per sample – FISH – biologist
Consumable costs – Droplet Digital PCR
Personnel time per sample – Sanger sequencing – technician
Personnel time per sample – Droplet Digital PCR – technician
Annual income – anatomopathologist
Personnel time per sample – FISH – pathologist
Personnel time per sample – FISH – technician
% equipment use – FISH
Personnel time per sample – IHC – technician
-30
-20
-10
-
10
20
30
€, 000s
€147,409
-20%
+20%
Center 4
Consumable costs – FISH
Consumable costs – IHC
Consumable costs – automated real-time PCR (Biocartis IDYLLA, Cobas)
Annual income – biologist
% equipment use – PANEL 3
Equipment purchase cost – PANEL 3
Personnel time per sample – automated real-time PCR (Biocartis IDYLLA, Cobas) – biologist
% equipment use – FISH
Equipment purchase cost – FISH
Annual income – anatomopathologist
% equipment use – automated real-time PCR (Biocartis IDYLLA, Cobas)
Equipment purchase cost – automated real-time PCR (Biocartis IDYLLA, Cobas)
Personnel time per sample – FISH – biologist
Personnel time per sample – automated real-time PCR (Biocartis IDYLLA, Cobas) – pathologist
Personnel time per sample – IHC – pathologist
-60
-40
-20
-
20
40
60
€, 000s
€287,555
Center 5
Consumable costs – PANEL 2
Consumable costs – PANEL 1
Consumable costs – Sanger sequencing
Consumable costs – IHC
Consumable costs – FISH
Annual income – biologist
Annual income – anatomopathologist
Personnel time per sample – Sanger sequencing – biologist
Personnel time per sample – Sanger sequencing – pathologist
Personnel time per sample – IHC – pathologist
Personnel time per sample – FISH – biologist
Personnel time per sample – FISH – pathologist
Personnel time per sample – Sanger sequencing – technician
Personnel time per sample – TSO500 – technician
Personnel time per sample – IHC – biologist
-300
-200
-100
-
100
200
300
€, 000s
–€440,782

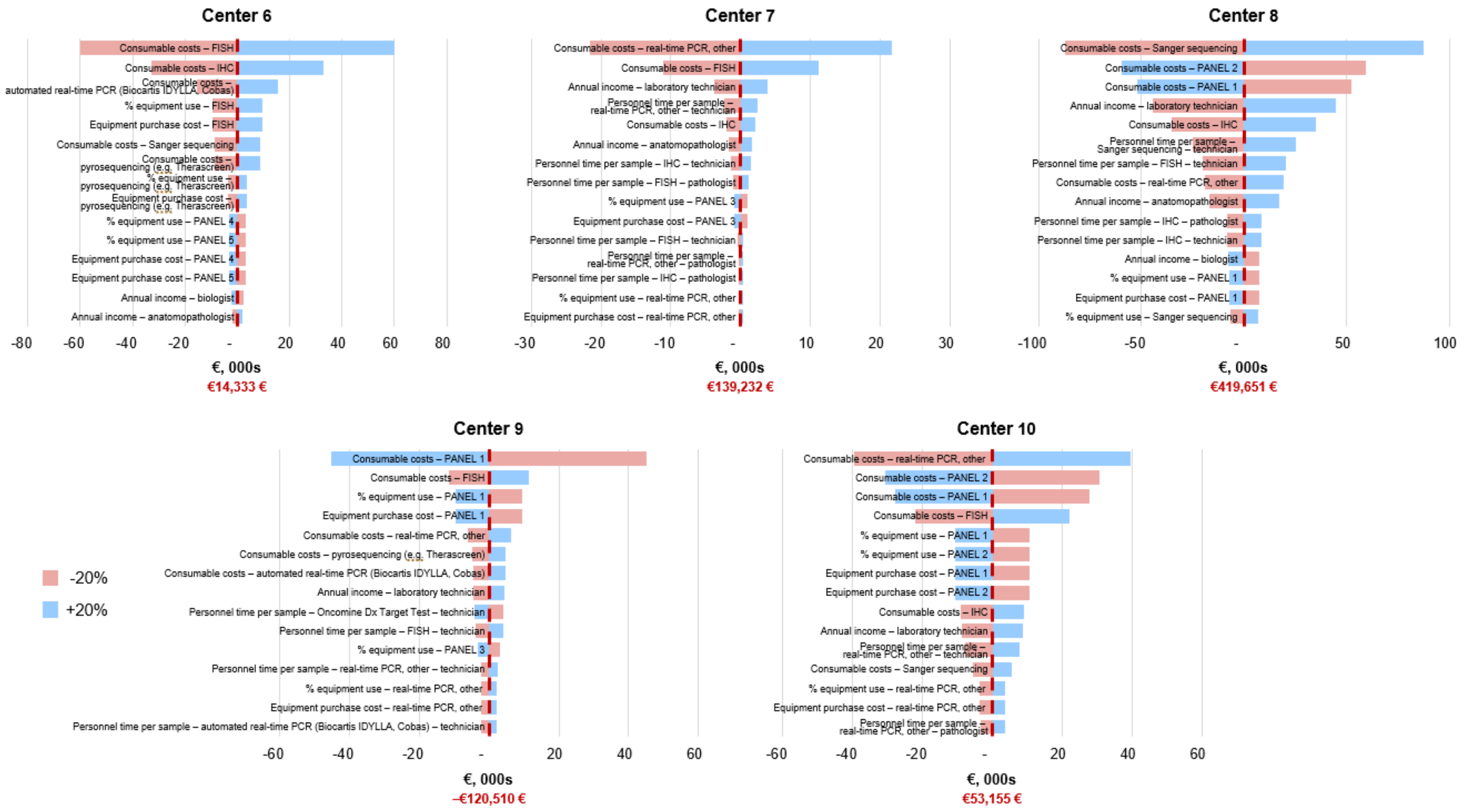


Values in red represent the annual difference in total costs between NGS and SGT per center, with positive values representing a cost saving for NGS versus SGT, and negative values representing a cost saving for SGT versus NGS. Each bar represents the impact on the difference in cost between the NGS and SGT of increasing (blue) or reducing (red) the cost of an individual cost parameter by 20%, in descending order of impact.

CP, Current Practice; FH, Future Horizons; FISH, fluorescence in situ hybridization; IHC, immunohistochemistry; ISH, in situ hybridization; PCR, polymerase chain reaction; SP, Starting Point; TSO, TruSight Oncology; UMI, unique molecular index.

## Supplementary tables

**Table S1.** Abbreviations.

| | |
|---|---|
| aNSCLC | Advanced non-small cell lung cancer |
| CP | Current Practice |
| CGP | Comprehensive genomic profiling |
| DDR | DNA damage response |
| DSA | Deterministic sensitivity analysis |
| EMA | European Medicines Agency |
| ESMO | European Society for Medical Oncology |
| FH | Future Horizons |
| FISH | Fluorescence in situ hybridization |
| IHC | Immunohistochemistry |
| ISH | In situ hybridization |
| MMRC | Mismatch repair |
| MSI | Microsatellite instability |
| NCCN | National Comprehensive Cancer Network |
| NGS | Next-generation sequencing |
| NSCLC | Non-small cell lung cancer |
| OCA | Oncomine™ Comprehensive Assay |
| OFA | Oncomine™ Focus Assay |
| OPA | Oncomine™ Precision Assay |
| PCR | Polymerase chain reaction |
| PD-L1 | Programmed cell death ligand 1 |
| RT | Real-time |
| SGT | Single gene testing |
| SP | Starting Point |
| TSO | TruSight Oncology |
| UMI | Unique molecular index |
| WES | Whole-exome sequencing |
| WGS | Whole-genome sequencing |

**Table S2.** Biomarkers tested in the real-world model.

| Biomarker | Center 1 | | | Center 2 | | | Center 3 | | | Center 4 | | | Center 5 | | | Center 6 | | | Center 7 | | | Center 8 | | | Center 9 | | | Center 10 | | | Total number of centers, n | | |
|---|---|---|---|---|---|---|---|---|---|---|---|---|---|---|---|---|---|---|---|---|---|---|---|---|---|---|---|---|---|---|---|---|---|
| | SP | CP | FH | SP | CP | FH | SP | CP | FH | SP | CP | FH | SP | CP | FH | SP | CP | FH | SP | CP | FH | SP | CP | FH | SP | CP | FH | SP | CP | FH | SP | CP | FH |
| *AKT* | ✔ | ✔ | ✔ | | | ✔ | ✔ | ✔ | ✔ | | | | | ✔ | ✔ | | | ✔ | | | ✔ | | ✔ | ✔ | | | 20% | 22% | 50% | ✔ | **3** | **5** | **9** |
| *ALK* | ✔ | ✔ | ✔ | ✔ | ✔ | ✔ | ✔ | ✔ | ✔ | ✔ | ✔ | ✔ | ✔ | ✔ | ✔ | 95% | ✔ | ✔ | ✔ | ✔ | ✔ | ✔ | ✔ | ✔ | ✔ | ✔ | ✔ | 90% | ✔ | ✔ | **10** | **10** | **10** |
| *BRAF* p.V600E | ✔ | ✔ | ✔ | ✔ | ✔ | ✔ | ✔ | ✔ | ✔ | ✔ | ✔ | ✔ | ✔ | ✔ | ✔ | 90% | ✔ | ✔ | 8% | ✔ | ✔ | ✔ | ✔ | ✔ | ✔ | ✔ | ✔ | 10% | 20% | ✔ | **10** | **10** | **10** |
| *BRCA 1,2* | | ✔ | ✔ | | | ✔ | | | ✔ | | | ✔ | | | ✔ | | | ✔ | | | ✔ | | ✔ | ✔ | | | 20% | | | | **0** | **2** | **9** |
| CGP | | ✔ | ✔ | | | ✔ | | | ✔ | | | ✔ | | | ✔ | | | | | | ✔ | | | ✔ | | | 20% | | | | **0** | **1** | **8** |
| DDR | | ✔ | ✔ | | | ✔ | | | ✔ | | | ✔ | | | ✔ | | | ✔ | | | ✔ | | | ✔ | | | 20% | 22% | 50% | ✔ | **1** | **2** | **10** |
| *EGFR* ex. 18,19,21 | ✔ | ✔ | ✔ | ✔ | ✔ | ✔ | ✔ | ✔ | ✔ | ✔ | ✔ | ✔ | ✔ | ✔ | ✔ | ✔ | ✔ | ✔ | ✔ | ✔ | ✔ | ✔ | ✔ | ✔ | ✔ | ✔ | ✔ | ✔ | ✔ | ✔ | **10** | **10** | **10** |
| *EGFR* ex. 20 | ✔ | ✔ | ✔ | ✔ | ✔ | ✔ | ✔ | ✔ | ✔ | ✔ | ✔ | ✔ | ✔ | ✔ | ✔ | ✔ | ✔ | ✔ | ✔ | ✔ | ✔ | ✔ | ✔ | ✔ | ✔ | ✔ | ✔ | 22% | 50% | ✔ | **10** | **10** | **10** |
| *ERBB2*/*HER2* | ✔ | ✔ | ✔ | ✔ | ✔ | ✔ | ✔ | ✔ | ✔ | | | ✔ | ✔ | ✔ | ✔ | 10% | ✔ | ✔ | | ✔ | ✔ | ✔ | ✔ | ✔ | | | 20% | 22% | 50% | ✔ | **7** | **8** | **10** |
| Pan-*FGFR* | ✔ | ✔ | ✔ | | | ✔ | ✔ | ✔ | ✔ | | | ✔ | | | ✔ | | | ✔ | | | ✔ | | | ✔ | | | 20% | 22% | 50% | ✔ | **3** | **3** | **10** |
| *HRAS* | ✔ | ✔ | ✔ | | | ✔ | ✔ | ✔ | ✔ | | | | | | ✔ | | | ✔ | | | ✔ | | | ✔ | | | 20% | | | | **2** | **2** | **8** |
| *JAK2/3* | ✔ | ✔ | ✔ | | | ✔ | | ✔ | ✔ | | | ✔ | | ✔ | ✔ | | | ✔ | | | ✔ | | | ✔ | | | 20% | | | | **1** | **3** | **9** |
| *KEAP1* | ✔ | ✔ | ✔ | | | ✔ | | ✔ | ✔ | | | ✔ | | ✔ | ✔ | | 50% | ✔ | | | ✔ | | | ✔ | | | 20% | | | | **1** | **4** | **9** |
| *KRAS* p.G12C | ✔ | ✔ | ✔ | ✔ | ✔ | ✔ | ✔ | ✔ | ✔ | ✔ | ✔ | ✔ | ✔ | ✔ | ✔ | 70% | ✔ | ✔ | 8% | ✔ | ✔ | ✔ | ✔ | ✔ | ✔ | ✔ | ✔ | 22% | 50% | ✔ | **10** | **10** | **10** |
| *MET* amp | ✔ | ✔ | ✔ | ✔ | ✔ | ✔ | ✔ | ✔ | ✔ | ✔ | ✔ | ✔ | 30% | ✔ | ✔ | | ✔ | ✔ | | ✔ | ✔ | ✔ | ✔ | ✔ | ✔ | ✔ | ✔ | 22% | 50% | ✔ | **8** | **10** | **10** |
| *MET* exon 14 skip | ✔ | ✔ | ✔ | ✔ | ✔ | ✔ | ✔ | ✔ | ✔ | ✔ | ✔ | ✔ | ✔ | ✔ | ✔ | | ✔ | ✔ | 8% | ✔ | ✔ | ✔ | ✔ | ✔ | ✔ | ✔ | ✔ | 22% | 50% | ✔ | **9** | **10** | **10** |
| MMR/MSI | | ✔ | ✔ | | | ✔ | | | ✔ | | | ✔ | | | ✔ | 10% | 10% | ✔ | | | ✔ | | ✔ | ✔ | | ✔ | ✔ | | | | **1** | **4** | **9** |
| *NRAS* | ✔ | ✔ | ✔ | | | ✔ | ✔ | ✔ | ✔ | | | | | | ✔ | | ✔ | ✔ | 8% | | ✔ | ✔ | ✔ | ✔ | | | 20% | 22% | 50% | ✔ | **5** | **5** | **9** |
| *NRG1* | ✔ | ✔ | ✔ | | ✔ | ✔ | | | ✔ | | | ✔ | ✔ | ✔ | ✔ | | | ✔ | | | ✔ | | ✔ | ✔ | | | 20% | | | | **2** | **4** | **9** |
| *NTRK1/2/3* | ✔ | ✔ | ✔ | ✔ | ✔ | ✔ | ✔ | ✔ | ✔ | ✔ | ✔ | ✔ | ✔ | ✔ | ✔ | 10% | ✔ | ✔ | 8% | ✔ | ✔ | | ✔ | ✔ | ✔ | ✔ | ✔ | 22% | 50% | ✔ | **9** | **10** | **10** |
| PD-L1 | ✔ | ✔ | ✔ | ✔ | ✔ | ✔ | ✔ | ✔ | ✔ | ✔ | ✔ | ✔ | ✔ | ✔ | ✔ | ✔ | ✔ | ✔ | ✔ | ✔ | ✔ | ✔ | ✔ | ✔ | ✔ | ✔ | ✔ | 74% | ✔ | ✔ | **10** | **10** | **10** |
| *PIK3CA* | | | | | | ✔ | ✔ | ✔ | ✔ | | | | | | | | | | | | | ✔ | ✔ | ✔ | | ✔ | ✔ | | | | **2** | **3** | **4** |
| *RET* | ✔ | ✔ | ✔ | ✔ | ✔ | ✔ | ✔ | ✔ | ✔ | ✔ | ✔ | ✔ | 60% | ✔ | ✔ | 10% | ✔ | ✔ | 8% | ✔ | ✔ | ✔ | ✔ | ✔ | ✔ | ✔ | ✔ | 22% | 50% | ✔ | **10** | **10** | **10** |
| *ROS1* | ✔ | ✔ | ✔ | ✔ | ✔ | ✔ | ✔ | ✔ | ✔ | ✔ | ✔ | ✔ | ✔ | ✔ | ✔ | 50% | ✔ | ✔ | ✔ | ✔ | ✔ | ✔ | ✔ | ✔ | ✔ | ✔ | ✔ | 73% | ✔ | ✔ | **10** | **10** | **10** |
| *STK11* | ✔ | ✔ | ✔ | | | ✔ | | ✔ | ✔ | | | ✔ | | ✔ | ✔ | | 50% | ✔ | | | ✔ | | ✔ | ✔ | | | 20% | | | | **1** | **5** | **9** |
| TMB | | ✔ | ✔ | | | ✔ | | | ✔ | | | ✔ | | | ✔ | | | 50% | | | ✔ | | ✔ | ✔ | | 5% | 20% | | | | **0** | **3** | **9** |
| *TP53* | ✔ | ✔ | ✔ | | | ✔ | | ✔ | ✔ | | | ✔ | | ✔ | ✔ | | 50% | ✔ | | | ✔ | ✔ | ✔ | ✔ | | | 20% | 22% | 50% | ✔ | **3** | **6** | **10** |
| Others | | | ✔ | | | | | | | | | | | | | | | | | | | | | | | | | | | | **0** | **0** | **1** |
| **Total number of clinically considered biomarkers, n** | | | | | | | | | | | | | | | | | | | | | | | | | | | | | | | | | |
| | **21** | **26** | **27** | **12** | **13** | **27** | **17** | **21** | **27** | **11** | **11** | **23** | **13** | **18** | **26** | **11** | **17** | **25** | **11** | **12** | **26** | **14** | **21** | **27** | **11** | **14** | **27** | **17** | **17** | **17** | | | |

Percentage values indicate the proportions of cases tested for each biomarker. Tick marks indicate that 100% of center cases were tested for a given biomarker.

The mean number of clinically considered biomarkers across centers was 14 in SP, 17 in CP, and 25 in FH.

*AKT*, protein kinase B; *ALK*, anaplastic lymphoma kinase; *BRAF*, v-raf murine sarcoma viral oncogene homolog B1; *BRCA*, BReast CAncer gene; CGP, comprehensive genomic profiling; CP, Current Practice; DDR, DNA damage response; *EGFR*, epidermal growth factor receptor; *ERBB2*, erb-b2 receptor tyrosine kinase 2; *FGFR*, fibroblast growth factor receptor; FH, Future Horizons; *HER2*, human epidermal growth factor receptor 2; *HRAS*, Harvey rat sarcoma viral oncogene homolog; *JAK*, Janus kinase; *KEAP1*, Kelch-like ECH-associated protein 1; *KRAS*, Kirsten rat sarcoma viral

oncogene homologue; *MET*, mesenchymal epithelial transition factor receptor; MMR, mismatch repair; MSI, microsatellite instability; *NRAS*, neuroblastoma RAS viral oncogene homolog; *NRG1*, neuregulin 1; *NTRK*, Neurotrophic tyrosine receptor kinase; PCR, polymerase chain reaction; PD-L1, programmed cell death ligand 1; *PIK3CA*, phosphatidylinositol-4,5-bisphosphate 3-kinase, catalytic subunit alpha; SP, Starting Point; *RET*, rearranged during transfection; *ROS1*, c-ros oncogene 1; RT, real-time; *STK11*, serine/threonine kinase 11; *TP53*, tumor protein p53.

**Table S3.** SGT techniques used in the real-world model.

| Biomarker | Center 1 | Center 2 | Center 3 | Center 4 | Center 5 | Center 6 | Center 7 | Center 8 | Center 9 | Center 10 |
|---|---|---|---|---|---|---|---|---|---|---|
| ***AKT*** | Sanger sequencing | - | - | - | - | - | - | Sanger sequencing | - | - |
| ***ALK*** | IHC | Idylla™ Multiplex and IHC | IHC (90%); FISH (10%) | Idylla™ Multiplex | IHC | IHC | IHC | FISH | FISH | IHC (93%); FISH (7%) |
| ***BRAF* p.V600E** | Sanger sequencing | IHC | Automated RT PCR (90%); Droplet Digital PCR (10%) | Automated RT PCR | Sanger sequencing | Pyrosequencin g | RT PCR (non-automated) | RT PCR (non-automated) | IHC | Sanger sequencing |
| ***BRCA1,2*** | Sanger sequencing | - | - | - | - | - | - | Sanger sequencing | - | - |
| ***EGFR* exons 18, 19, 21** | Sanger sequencing | Automated RT PCR | Automated RT PCR (90%); Droplet Digital PCR (10%) | Automated RT PCR | Sanger sequencing | Automated RT PCR | RT PCR (non-automated) | Sanger sequencing (50%); Automated RT PCR (50%) | Automated RT PCR (80%); Droplet Digital PCR (20%) | Automated RT PCR |
| ***EGFR* exon 20** | Sanger sequencing | - | Automated RT PCR (90%); Droplet Digital PCR (10%) | - | Sanger sequencing | Sanger sequencing | RT PCR (non-automated) | Sanger sequencing (50%); Automated RT PCR (50%) | RT PCR (non-automated) | Automated RT PCR (67%); Droplet Digital PCR (33%) |
| ***ERBB2/ HER2*** | Sanger sequencing | RT PCR (non-automated) | - | - | - | Sanger sequencing | - | Sanger sequencing (50%); IHC (50%) | - | - |
| **Pan-*FGFR*** | - | - | - | - | - | - | - | - | - | - |
| ***HRAS*** | Sanger sequencing | - | - | - | - | - | - | - | - | - |
| ***JAK2/3*** | Sanger sequencing | - | - | - | - | - | - | - | - | - |
| ***KEAP1*** | Sanger sequencing | - | - | - | - | - | - | - | - | - |
| ***KRAS* p.G12C** | Sanger sequencing | Automated RT PCR | Automated RT PCR (90%); Droplet Digital PCR (10%) | Automated RT PCR | Sanger sequencing | Pyrosequencin g | RT PCR (non-automated) | Sanger sequencing (50%); RT PCR (non-automated) (50%) | RT PCR (non-automated) | - |
| ***MET* amplification** | ISH | FISH | FISH | IHC (50%); FISH (50%) | - | ISH | - | FISH | FISH | - |

| Biomarker | Center 1 | Center 2 | Center 3 | Center 4 | Center 5 | Center 6 | Center 7 | Center 8 | Center 9 | Center 10 |
|---|---|---|---|---|---|---|---|---|---|---|
| ***MET* exon 14 skipping** | Sanger sequencing | Idylla™ Multiplex | Automated RT PCR (90%); Droplet Digital PCR (10%) | Idylla™ Multiplex | - | Automated RT PCR | - | Sanger sequencing | Pyrosequencin g | - |
| **MMR/MSI** | - | - | - | - | - | Automated RT PCR | - | Sanger sequencing | Sanger sequencing | - |
| ***NRAS*** | Sanger sequencing. | - | Automated RT PCR (90%); Droplet Digital PCR (10%) | - | - | Pyrosequencin g | RT PCR (non-automated) | Sanger sequencing | - | - |
| ***NRG1*** | - | - | - | - | - | - | - | FISH | - | - |
| ***NTRK1/2/3*** | FISH | Idylla™ Multiplex | FISH (90%); IHC (10%) | Automated RT PCR | - | IHC | RT PCR (non-automated) | FISH | IHC | IHC |
| **PD-L1** | IHC | IHC | IHC | IHC | IHC | IHC | IHC | IHC | IHC | IHC |
| ***PIK3CA*** | - | - | Automated RT PCR (90%); Droplet Digital PCR (10%) | - | - | - | - | Sanger sequencing | RT PCR (non-automated) | - |
| ***RET*** | FISH | Idylla™ Multiplex | FISH | Idylla™ Multiplex | FISH | Automated RT PCR | - | FISH | FISH | FISH |
| ***ROS1*** | FISH | Idylla™ Multiplex and IHC | IHC (90%); FISH (10%) | Idylla™ Multiplex | FISH | ISH | IHC | FISH | RT PCR (non-automated) | IHC (92%); FISH (8%) |
| ***STK11*** | Sanger sequencing | - | - | - | - | - | - | Sanger sequencing | - | - |
| ***TP53*** | Sanger sequencing | - | - | - | - | Sanger sequencing | - | Sanger sequencing | - | IHC |

SGT-based testing techniques were the same in the SP and CP scenarios for each center. In the FH scenario, SGT was performed for PD-L1 only.

Where unspecified, technique use is 100%.

*AKT*, protein kinase B; *ALK*, anaplastic lymphoma kinase; *BRAF*, v-raf murine sarcoma viral oncogene homolog B1; *BRCA*, BReast CAncer gene; CP, Current Practice; *EGFR*, epidermal growth factor receptor; *ERBB2*, erb-b2 receptor tyrosine kinase 2; *FGFR*, fibroblast growth factor receptor; FH, Future Horizons; FISH, fluorescence in situ hybridization; *HER2*, human epidermal growth factor receptor 2; *HRAS*, Harvey rat sarcoma viral oncogene homolog; IHC, immunohistochemistry; ISH, in situ hybridization; *JAK*, Janus kinase; *KEAP1*, Kelch-like ECH-associated protein 1; *KRAS*, Kirsten rat sarcoma viral oncogene homologue; *MET*, mesenchymal epithelial transition factor receptor; MMR, mismatch repair; MSI, microsatellite instability; *NRAS*, neuroblastoma RAS viral oncogene homolog; *NRG1*, neuregulin 1; PCR, polymerase chain reaction; *NTRK*, Neurotrophic tyrosine receptor kinase; PD-L1, programmed cell death ligand 1; *PIK3CA*, phosphatidylinositol-4,5-bisphosphate 3-kinase, catalytic subunit alpha; *RET*, rearranged during transfection; *ROS1*, c-ros oncogene 1; RT, real-time; SGT, single gene testing; SP, Starting Point; *STK11*, serine/threonine kinase 11; *TP53*, tumor protein p53.

**Table S4.** NGS panels used in the real-world model.

| | | Center 1 | Center 2 | Center 3 | Center 4 | Center 5 | Center 6 | Center 7 | Center 8 | Center 9 | Center 10 |
|---|---|---|---|---|---|---|---|---|---|---|---|
| **Panel 1** | **Genetic material** | DNA | DNA | DNA/RNA | DNA/RNA | DNA | DNA | RNA | DNA | DNA/RNA | DNA |
| | **No. of biomarkers** | 53 | 50 | 52 | 12 | 523 | 25 | 17 | 30 | 23 | 19 |
| | **Manufacturer / model** | Thermo Fisher Scientific / HDonco (custom) | Thermo Fisher Scientific / OPA DNA | Thermo Fisher Scientific / OFA | Thermo Fisher Scientific / Custom panel SiRe® | Illumina / TSO500 | Thermo Fisher Scientific / Ion Torrent Ion AmpliSeq colon and lung research panel v2 | Archer / FusionPlex Lung v2 | Thermo Fisher Scientific / Customized panel | Thermo Fisher Scientific / Oncomine Dx Target Test | QIAGEN / GeneRead QIAact Lung DNA UMI Panel |
| | **Scenario** | | | | | | | | | | |
| | **SP** | 90% | 100% | 100% | 100% | 100% | 50% | 100% | 100% | 100% | 100% |
| | **CP** | - | 100% | - | - | 90% | - | - | 100% | 100% | 100% |
| | **FH** | - | - | - | - | - | - | - | - | 100% | - |
| **Panel 2** | **Genetic material** | RNA | RNA | - | - | RNA | RNA | DNA | RNA | DNA/RNA | RNA |
| | **No. of biomarkers** | 17 | 50 | | | 17 | 5 | 83 | 51 | 500 | 79 |
| | **Manufacturer / model** | Archer / FusionPlex Lung v2 | Thermo Fisher Scientific / OPA RNA | | | Archer / FusionPlex Lung v2 | Thermo Fisher Scientific / Ion Torrent Ion AmpliSeq RNA fusion Lung cancer panel extended (+NTRK2/3, FGFR1/2/3) | Roche / Custom panel 1 | Thermo Fisher Scientific / Oncomine Comprehensiv e v3 RNA | Thermo Fisher Scientific / Oncomine Comprehensiv e plus | QIAGEN / GeneRead QIAact Lung RNA UMI Panel |
| | **Scenario** | | | | | | | | | | |
| | **SP** | 90% | 100% | | | 100% | 100% | 10% | - | 5% | 100% |
| | **CP** | - | 100% | | | 100% | - | - | 100% | - | 100% |
| | **FH** | - | - | | | - | - | - | - | - | - |
| **Panel 3** | **Genetic material** | DNA/RNA | DNA/RNA | DNA/RNA | DNA/RNA | DNA | DNA | DNA/RNA | DNA/RNA | DNA/RNA | DNA/RNA |
| | **No. of biomarkers** | DNA, 523; RNA, 55 | 161 | 161 | 50 | Genome-wide | 50 | 80 | 500+ | 830 | 830 |
| | **Manufacturer /model** | Illumina / TSO500 | Thermo Fisher Scientific / OCA v3 | Thermo Fisher Scientific / OCA v3 | Thermo Fisher Scientific / Extensive Custom Panel OPA | Illumina / WGS | Thermo Fisher Scientific / Ion Torrent Ion AmpliSeq cancer hot spot v2 | Roche / Custom panel 2 | Thermo Fisher Scientific / OCAplus or Illumina/TSO5 00 | QIAGEN / QIAseq multimodal HC | QIAGEN / QIAseq multimodal HC |
| | **Scenario** | | | | | | | | | | |
| | **SP** | 10% | 5% | - | - | - | 50% | - | - | - | - |
| | **CP** | 100% | 5% | 100% | 100% | - | - | 100% | - | 5% | - |
| | **FH** | - | - | - | - | 30% | - | 100% | 100% | 20% | 100% |
| **Panel 4** | **Genetic material** | DNA | DNA/RNA | - | - | RNA | DNA/RNA | - | - | - | - |
| | **No. of biomarkers** | ~25k | 321 | | | Genome-wide | 52 | | | | |

| | | Center 1 | Center 2 | Center 3 | Center 4 | Center 5 | Center 6 | Center 7 | Center 8 | Center 9 | Center 10 |
|---|---|---|---|---|---|---|---|---|---|---|---|
| | **Manufacturer / model** | Twist / Exome v2 | Illumina / Next-Seq Dx | | | Illumina / RNAseq | Thermo Fisher Scientific / OFA | | | | |
| | **Scenario** | | | | | | | | | | |
| | **SP** | - | - | | | - | - | | | | |
| | **CP** | - | - | | | - | 100% | | | | |
| | **FH** | 100% | 100% | | | 70% | - | | | | |
| **Panel 5** | **Genetic material** | RNA | - | DNA/RNA | DNA/RNA | DNA/RNA | - | - | - | - | - |
| | **No. of biomarkers** | ~25k | | DNA, 523; RNA, 55 | 500+ | 161 | | | | | |
| | **Manufacturer / model** | Illumina / Ribo-Zero Plus rRNA Depletion Kit | | Illumina / TSO500 | Thermo Fisher Scientific / OCA plus v3, Extensive Custom Panel | Thermo Fisher Scientific / OCAv3 | | | | | |
| | **Scenario** | | | | | | | | | | |
| | **SP** | - | | - | - | - | | | | | |
| | **CP** | - | | - | - | 10% | | | | | |
| | **FH** | 100% | | 100% | 100% | - | | | | | |
| **Panel 6** | **Genetic material** | - | - | - | - | DNA | DNA/RNA | - | - | - | - |
| | **No. of biomarkers** | | | | | Whole exome | 60 | | | | |
| | **Manufacturer / model** | | | | | Illumina / WES | Thermo Fisher Scientific / Ion Torrent Ion AmpliSeq HD panel (custom) | | | | |
| | **Scenario** | | | | | | | | | | |
| | **SP** | | | | | - | - | | | | |
| | **CP** | | | | | - | - | | | | |
| | **FH** | | | | | 70% | 100% | | | | |

CP, Current Practice; FH, Future Horizons; NGS, next-generation sequencing; OCA, Oncomine™ Comprehensive Assay; OFA, Oncomine™ Focus Assay; OPA, Oncomine™ Precision Assay; SP, Starting Point; TSO, TruSight Oncology; UMI, unique molecular index; WES, whole-exome sequencing; WGS, whole-genome sequencing.

**Table S5.** Minimum number of biomarkers required for per-patient cost savings for NGS versus SGT in the standardized model.

| Center | Minimum number of biomarkers for costs per patient NGS <SGT | |
|---|---|---|
| | SP | CP |
| 1 | 10 | 8 |
| 2 | 15 | 15 |
| 3 | 7 | 9 |
| 4 | 5 | 7 |
| 5 | 12 | 13 |
| 6 | 7 | 12 |
| 7 | 6 | 4 |
| 8 | 9 | 18 |
| 9 | 13 | 18 |
| 10 | 12 | 10 |
| Mean | 10 | 12 |

CP, Current Practice; NGS, next-generation sequencing; SGT, single-gene testing; SP, Starting Point.

**Table S6.** Effect of varying the most impactful cost parameter by ±20% on total annual testing cost differences for NGS versus SGT in (A) the real-world model, and (B) the standardized model.

(A)

| | **Total annual testing costs** | | | | | |
|---|---|---|---|---|---|---|
| | **SP** | | | **CP** | | |
| **Original analysis** | NGS <SGT | NGS >SGT | NGS <SGT | NGS <SGT | NGS >SGT | NGS <SGT |
| **Alteration of most impactful cost parameter by ±20%** | NGS <SGT | NGS >SGT | NGS >SGT | NGS <SGT | NGS >SGT | NGS >SGT |
| Center | | | | | | |
| 1 | x | | | x | | |
| 2 | | x | | | x | |
| 3 | x | | | x | | |
| 4 | x | | | x | | |
| 5 | | | x | x | | |
| 6 | | x | | x | | |
| 7 | | x | | x | | |
| 8 | x | | | x | | |
| 9 | | x | | | x | |
| 10 | | x | | | x | |
| **Total** | **4** | **5** | **1** | **7** | **3** | **0** |

(B)

| | **Total annual testing costs** | | | | | |
|---|---|---|---|---|---|---|
| | **SP** | | | **CP** | | |
| **Original analysis** | NGS <SGT | NGS >SGT | NGS <SGT | NGS <SGT | NGS >SGT | NGS <SGT |
| **Alteration of most impactful cost parameter by ±20%** | NGS <SGT | NGS >SGT | NGS >SGT | NGS <SGT | NGS >SGT | NGS >SGT |
| Center | | | | | | |
| 1 | | x | | x | | |
| 2 | | x | | | x | |
| 3 | x | | | x | | |
| 4 | x | | | x | | |
| 5 | | x | | | x | |
| 6 | x | | | | | x |
| 7 | x | | | x | | |
| 8 | | x | | x | | |
| 9 | | x | | | x | |
| 10 | | x | | x | | |
| **Total** | **4** | **6** | **0** | **6** | **3** | **1** |

CP, Current Practice; NGS, next-generation sequencing; SGT, single gene testing; SP, Starting Point.
